# Blocking skeletal muscle DHPRs/Ryr1 prevents neuromuscular synapse loss in mutant mice deficient in type III Neuregulin 1 (CRD-Nrg1)

**DOI:** 10.1371/journal.pgen.1007857

**Published:** 2019-03-14

**Authors:** Yun Liu, Yoshie Sugiura, Fujun Chen, Kuo-Fen Lee, Qiaohong Ye, Weichun Lin

**Affiliations:** 1 Department of Neuroscience, UT Southwestern Medical Center, Dallas, United States of America; 2 The Salk Institute, La Jolla, United States of America; The Jackson Laboratory, UNITED STATES

## Abstract

Schwann cells are integral components of vertebrate neuromuscular synapses; in their absence, pre-synaptic nerve terminals withdraw from post-synaptic muscles, leading to muscle denervation and synapse loss at the developing neuromuscular junction (NMJ). Here, we report a rescue of muscle denervation and neuromuscular synapses loss in type III Neuregulin 1 mutant mice (*CRD-Nrg1*^−/−^), which lack Schwann cells. We found that muscle denervation and neuromuscular synapse loss were prevented in *CRD-Nrg1*^−/−^mice when presynaptic activity was blocked by ablating a specific gene, such as *Snap25* (synaptosomal-associated 25 kDa protein) or *Chat* (choline acetyltransferase). Further, these effects were mediated by a pathway that requires postsynaptic acetylcholine receptors (AChRs), because ablating *Chrna1* (acetylcholine receptor α1 subunit), which encodes muscle-specific AChRs in *CRD-Nrg1*^−/−^mice also rescued muscle denervation. Moreover, genetically ablating muscle dihydropyridine receptor (DHPR) β1 subunit (*Cacnb1*) or ryanodine receptor 1 (*Ryr1*) also rescued muscle denervation and neuromuscular synapse loss in *CRD-Nrg1*^−/−^mice. Thus, these genetic manipulations follow a pathway–from presynaptic to postsynaptic, and, ultimately to muscle activity mediated by DHPRs and Ryr1. Importantly, electrophysiological analyses reveal robust synaptic activity in the rescued, Schwann-cell deficient NMJs in *CRD-Nrg1*^−/−^*Cacnb1*^−/−^or *CRD-Nrg1*^−/−^*Ryr1*^−/−^mutant mice. Thus, a blockade of synaptic activity, although sufficient, is not necessary to preserve NMJs that lack Schwann cells. Instead, a blockade of muscle activity mediated by DHRPs and Ryr1 is both necessary and sufficient for preserving NMJs that lack Schwann cells. These findings suggest that muscle activity mediated by DHPRs/Ryr1 may destabilize developing NMJs and that Schwann cells play crucial roles in counteracting such a destabilizing activity to preserve neuromuscular synapses during development.

## Author summary

Nerve cells (neurons) communicate with each other through specialized focal connections called synapses. Synapses are commonly assembled into a tripartite structure composed of pre- and post-synaptic neurons, and a glial cell that is closely associated with both pre- and post-synaptic components. At the vertebrate neuromuscular synapse (the neuromuscular junction, NMJ), the tripartite structure is composed of a nerve terminal, a muscle fiber and a glial cell called the terminal Schwann cell. Previous studies have shown that Schwann cells are vital to the NMJ–without Schwann cells, the NMJ fails to form. Here, we report unexpected findings that bipartite NMJs can be established *in vivo*, in the absence of Schwann cells. These results were accomplished by ablating specific genes in mice, ultimately resulting in a total blockade of muscle activity. Thus, Schwann cells become dispensable for the NMJ if muscle activity is blocked. These findings suggest that muscle activity normally destabilizes the NMJ during synapse formation and that Schwann cells play crucial roles in NMJ formation by opposing this muscle-derived destabilizing activity in order to preserve the developing neuromuscular synapses.

## Introduction

Like all chemical synapses in the brain, the NMJ–the synaptic connections between motor neurons and skeletal muscles–is assembled as a tripartite structure that includes a presynaptic motor nerve terminal, a postsynaptic muscle cell and a terminal Schwann cell, which caps the motor nerve terminal. These three cellular components interact to support the normal physiological function of the NMJ [1–10]. Importantly, Schwann cells are required for the maintenance of the NMJ [11–15] and play crucial roles in the re-establishment of the NMJs during nerve regeneration after injury [16–18]. Motor neurons in turn produce the glycoprotein neuregulin 1 (NRG1), predominantly as the cysteine-rich domain isoform of neuregulin 1 (type III NRG1, or CRD-NRG1) [19–21]. NRG1 interacts with receptor tyrosine kinase erbB receptors and play crucial roles in synapse formation and function [19, 22–35].

Emerging evidence suggests that NRG1/erbB expression is correlated with the state of skeletal muscle innervation/denervation [36]. Deficiencies in NRG1/erbB signaling–as shown in *CRD-Nrg1*^−/−^, *erbB2*^−/−^and *erbB3*^−/−^mutant mice–lead to a loss of Schwann cells and, consequently, a retraction of motor nerve terminals from diaphragm muscle, resulting in muscle denervation and neuromuscular synapse loss [37–43]. These defects are likely due to the loss of Schwann cells, rather than a loss of NRG1/erbB signaling from motor neurons to muscles, since deleting erbBs specifically in muscles does not affect NMJ formation and function [44]. How an absence of Schwann cells may cause muscle denervation and neuromuscular synapse loss, however, remains unclear.

Synaptic activity plays crucial roles in sculpting neural circuits [45, 46]. Terminal Schwann cells are known to play important roles in regulating synaptic activity at the NMJ [5, 47–49], suggesting possible relationships among Schwann cells, activity and synapse formation. In this study, we ask the question if an absence of specific activity at pre-synaptic nerve terminals, post-synaptic acetylcholine receptors (AChRs), or muscle fibers, may affect NMJ formation in the absence of Schwann cells. To address this question, we blocked pre-synaptic activity in *CRD-Nrg1*^−/−^mice, which lack Schwann cells, by ablating specific genes known to be required for transmitter release from the nerve terminal. Surprisingly, blocking neurotransmitter release results in a rescue of muscle denervation and prevents the neuromuscular synapse loss that normally occurred in *CRD-Nrg1*^−/−^mice. We further show that these effects were mediated by postsynaptic acetylcholine receptors (AChRs), because genetic elimination of muscle-specific AChRs in *CRD-Nrg1*^−/−^mice also rescued muscle denervation. And finally, we show that these effects were mediated through muscle activity because genetically ablating either dihydropyridine receptors (DHPRs) or ryanodine receptor 1 (Ryr1) also rescues muscle denervation and neuromuscular synapse loss in *CRD-Nrg1*^−/−^mice. Together, these results demonstrate that bipartite NMJs lacking Schwann cells can be established if muscle activity is blocked, suggesting that muscle activity mediated by DHPRs/Ryr1 plays a key role in preserving Schwann cell-deficient NMJs.

## Results

### Blocking pre-synaptic activity in *CRD-Nrg1*^*–/–*^mice prevents neuromuscular synapse loss

To investigate how synaptic activity might impact the development of neuromuscular synapses in *CRD-Nrg1*^*–/–*^mice, we took several genetic approaches. First, we took advantage of previously characterized mutant mice that lack pre-synaptic activity. For example, evoked synaptic transmission is completely blocked in mutant mice deficient in synaptosomal-associated 25 kDa protein (SNAP25) [50], a key protein component of Soluble N-ethylmaleimide-sensitive factor attachment protein receptors (SNAREs) complexes required for regulated exocytosis in all chemical synapses [51–55]. We therefore bred *CRD-Nrg1* mutant mice with *Snap25* mutant mice, and generated double mutant mice deficient in both *CRD-Nrg1* and *Snap25* (*CRD-Nrg1*^*–/–*^*Snap25*^*–/–*^).

**Fig 1:**
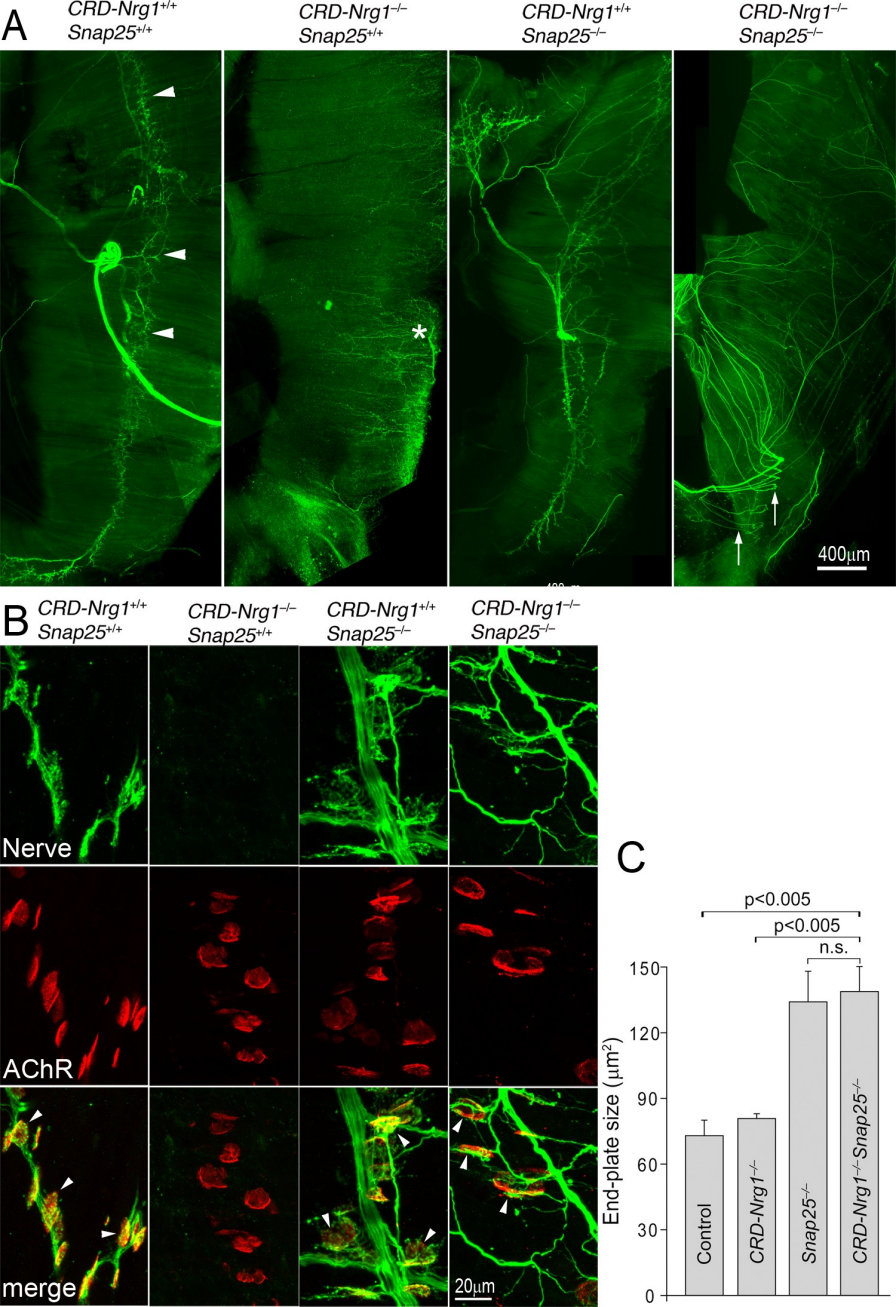
Blocking pre-synaptic activity prevents synapse loss in *CRD-Nrg1*^−/−^mice. Embryonic diaphragm muscles (E18.5) were immunostained with anti-NF150 and anti-Syt2 antibodies to reveal pre-synaptic nerves, and labelled with Texas Red conjugated α-bgt to mark post-synaptic endplates. **A**: Low power views of hemi-diaphragm muscles, showing the phrenic nerves trunk and nerve terminals. Arrowheads in wild-type muscle (*CRD-Nrg1*^*+/+*^*Snap25*^*+/+*^) point to the nerve terminals. In *CRD-Nrg1*^*–/–*^*Snap25*^*+/+*^, the phrenic nerve is absent (* indicates the sensory nerves projecting from the edge towards the center). In *CRD-Nrg1*^*–/–*^*Snap25*^*–/–*^muscle, the phrenic nerve innervation is rescued, and the nerves are highly defasciculated (arrows). **B**: High-power images showing individual neuromuscular synapses (arrowheads). Neuromuscular synapses are absent in *CRD-Nrg1*^*–/–*^*Snap25*^*+/+*^ but are present in *CRD-Nrg1*^*–/–*^*Snap25*^*–/–*^muscle (arrowheads). **C**: The average size of postsynaptic endplates in the diaphragm muscles is significantly increased in *CRD-Nrg1*^*–/–*^*Snap25*^*–/–*^mice (138.3 ± 11.4 μm^2^, n = 177, N = 3), compare with control (72.7 ± 7.0 μm^2^, n = 201, N = 3, P = 0.0039), or *CRD-Nrg1*^*–/–*^mice (80.5 ± 2.2 μm^2^, n = 287, N = 3, P = 0.0036), but not significantly different from those in *Snap25*^*–/–*^mice (133.6 ± 13.9 μm^2^, n = 198, N = 3, P = 0.7670). Scale bars: A: 400 μm; B: 20 μm. n: number of postsynaptic endplates; N: number of mice.

Surprisingly, in contrast to a loss of phrenic innervation in the diaphragm muscle in *CRD-Nrg1*^*–/–*^mice [38] (see also Fig 1A, second left panel), the diaphragm muscles in *CRD-Nrg1*^*–/–*^*Snap25*^*–/–*^mice were fully innervated, despite defasciculation (Fig 1A, first right panel). Immunostaining of the diaphragm muscles (E18.5) using a mixture of antibodies (anti-NF150 and anti-Syt2 antibodies) revealed the presence of phrenic innervation in wild-type (Fig 1A, *CRD-Nrg1*^*+/+*^*Snap25*^*+/+*^), but not *CRD-Nrg1*^*–/–*^*Snap25*^*+/+*^ mice (Fig 1A, second panel). In contrast, the diaphragm muscle in *CRD-Nrg1*^*–/–*^*Snap25*^*–/–*^mice was fully innervated, although the nerves appeared highly defasciculated (Fig 1A, right panel). More important, post-synaptic endplates labeled by Texas Red conjugated α-bgt were juxtaposed with presynaptic nerve terminals, indicating that the NMJs were established in the diaphragm muscles in *CRD-Nrg1*^*–/–*^*Snap25*^*–/–*^mice (Fig 1B). Furthermore, the average size of postsynaptic endplates in *CRD-Nrg1*^*–/–*^*Snap25*^*–/–*^mice was significantly larger than those in control or *CRD-Nrg1*^*–/–*^mice (Fig 1C).

These results indicated that muscle denervation and NMJ loss were rescued in *CRD-Nrg1* mutant mice that were also deficient in *Snap25* (*CRD-Nrg1*^*–/–*^*Snap25*^*–/–*^). At the NMJ, motor nerve terminals are known to co-release both acetylcholine (ACh) and adenosine 5′-triphosphate (ATP), which has been shown to regulate the expression and stability of post-synaptic AChRs [56–59]. Ablating Snap25 blocks vesicular exocytosis and thus blocks the release of both ACh and ATP. This raises the question whether the effects seen in *CRD-Nrg1*^*–/–*^*Snap25*^*–/–*^mice were due to a blockade of the release of ACh, ATP, or both.

**Fig 2:**
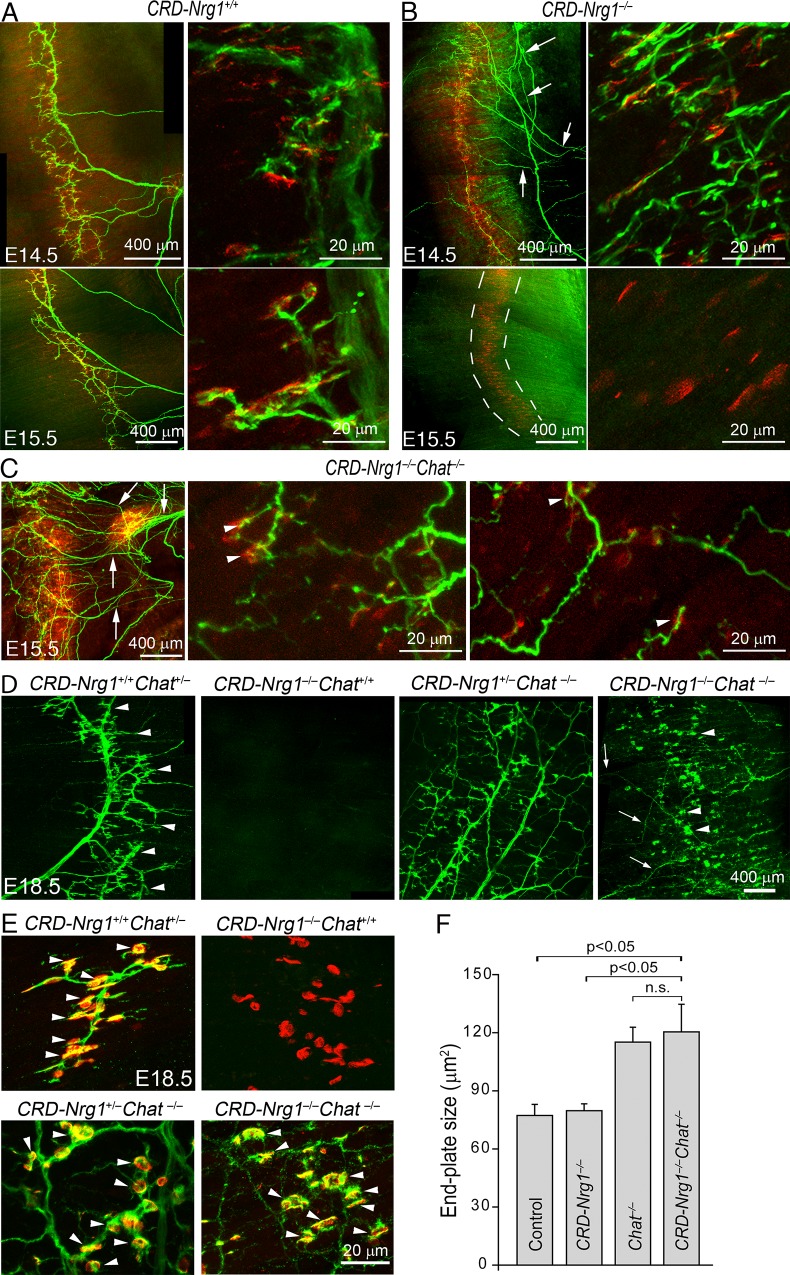
Blockade of cholinergic neurotransmission *(Chat−*^*/–*^*)* prevents synapse loss caused by *CRD-Nrg1* deficiency. **A-C:** Initial NMJ formation in *CRD-Nrg1*^*–/–*^and *CRD-Nrg1*^*–/–*^*Chat^−^*^/–^mice. Confocal images (low power, left panels; high power, right panels) of embryonic diaphragm muscles at E14.5 and E15.5. Presynaptic nerves (green) are revealed by immunostaining using a mixture of anti-NF150 and anti-Syt2 antibodies, and postsynaptic end-plates (red) by Texas Red-conjugated α-bungarotoxin. At E14.5, the diaphragm muscle in *CRD-Nrg1*^*–/–*^mice is innervated, but the nerves are highly defasciculated (arrows in B). However, at E15.5, the diaphragm muscle in *CRD-Nrg1*^*–/–*^mice is denervated, leaving the end-plates vacant (bordered by dashed lines). Interestingly, muscle denervation is fully rescued in *CRD-Nrg1*^*–/–*^*Chat^−^*^/–^mice. Similarly, the nerves are highly defasciculated (arrows in C). High-power images show formation of nascent neuromuscular synapses (arrowheads in C) on the right two panels in C. **D-E**: Confocal images of E18.5 whole-mount diaphragm muscles (D, low power; E, high power) labeled by anti-syntaxin1 antibodies (green) and Texas Red-conjugated α-bgt (red). In control mice (*CRD-Nrg1*^*+/+*^*Chat* ^*+/–*^), the nerve extends nerve branches (arrowheads in D) targeted to the central region of the muscle. The nerve is absent in *CRD-Nrg1*^*–/–*^*Chat* ^*+/+*^ mice. The lack of innervation is rescued in *CRD-Nrg1*^*–/–*^*Chat*^*−/−*^mice. The nerve branches are defasciculated (arrow in D, *CRD-Nrg1*^*–/–*^*Chat*^*−/−*^), but the nerve terminals are intensely labeled by anti-syntaxin1 antibodies (arrowheads in D, *CRD-Nrg1*^*–/–*^*Chat*^*−/−*^) and are localized to the central region of the muscle. High power images in E show nerve terminals juxtaposing with AChR clusters and form NMJs (arrowheads in E) in control (*CRD-Nrg1*^*+/+*^*Chat* ^*+/–*^), *Chat*^*−/−*^and *CRD-Nrg1*^*–/–*^*Chat*^*−/−*^muscles. However, nerve terminals are absent in *CRD-Nrg1*^*–/–*^*Chat* ^*+/+*^ muscle, leaving AChRs vacant. **F**: Quantitative analyses of postsynaptic endplate size in E18.5 diaphragm muscles. The average endplate size is 77.2 ± 5.7 μm^2^ in control (n = 450, N = 3), 79.7 ± 3.6 μm^2^ in *CRD-Nrg1*^*–/–*^(n = 313, N = 3), 115.1 ± 7.7 μm^2^ in *Chat*^−/−^(n = 576, N = 3) and 120.4 ± 14.3 μm^2^ in *CRD-Nrg1*^*–/–*^*Chat*^*−/−*^mice (n = 526, N = 3). The average endplate size in *CRD-Nrg1*^*–/–*^*Chat*^*−/−*^mice is significantly increased (P < 0.05) compared with control or *CRD-Nrg1*^*–/–*^mice. The endplate size is not statistically significant (n. s.) between *CRD-Nrg1*^*–/–*^*Chat*^*−/−*^and *Chat*^*–/–*^mice (one-way ANOVA, followed by Tukey test). N: number of mice; n: number of end-plates.

To address this question, we turned to mutant mice deficient in ChAT–the enzyme required for synthesizing ACh [60, 61]. We blocked cholinergic synaptic transmission in *CRD-Nrg1*^*–/–*^mice by generating double mutant mice deficient in both *CRD-Nrg1* and *Chat* (*CRD-Nrg1*^*–/–*^*Chat*^*−/−*^). As shown in Fig 2, the diaphragm muscles in *CRD-Nrg1*^*–/–*^mice were transiently innervated at E14.5 (Fig 2B) but completely denervated by E15.5 (Fig 2B). In contrast, diaphragm muscles in the *CRD-Nrg1*^*–/–*^mice that also lacked ChAT (*CRD-Nrg1*^*–/–*^*Chat*^*−/−*^) were fully innervated at E15.5 (Fig 2C), E16.5 (S1 Fig) and E18.5 (Fig 2D). Furthermore, the intramuscular nerves in *CRD-Nrg1*^*–/–*^(Fig 2B) and *CRD-Nrg1*^*–/–*^*Chat*^*−/−*^(Fig 2C and 2D) were highly defasciculated. Higher-power images further revealed that presynaptic nerve terminals were juxtaposed to postsynaptic AChR-enriched endplates in *CRD-Nrg1*^*–/–*^*Chat*^*−/−*^mice (Fig 2E). Similar to control mice, the apposition of pre-synaptic nerve terminals and post-synaptic end-plates in *CRD-Nrg1*^*–/–*^*Chat*^*−/−*^mice progressed throughout neuromuscular synaptogenesis (E15.5, E16.5 and E18.5) (Fig 2). We counted 526 postsynaptic endplates from the diaphragm muscles of 3 *CRD-Nrg1*^*–/–*^*Chat*^*−/−*^mice (E18.5); 100% of these 526 endplates were juxtaposed by presynaptic nerve terminals (Fig 2E). Furthermore, the endplates in *CRD-Nrg1*^*–/–*^*Chat*^*–/–*^or *Chat*^−/−^mice were noticeably larger than those in control or *CRD-Nrg1*^*–/–*^mice. For example, the average endplate sizes were 120.4 ± 14.3 μm^2^ in *CRD-Nrg1*^*–/–*^*Chat*^*−/−*^mice (n = 526, N = 3) and 115.1 ± 7.7 μm^2^ in *Chat*^−/−^(n = 576, N = 3), which represented an increase of more than 50% compared to the end-plates in either control (77.2 ± 5.7 μm^2^, n = 450, N = 3) or *CRD-Nrg1*^*–/–*^mice (79.7 ± 3.6 μm^2^, n = 313, N = 3) (Fig 2F).

In *CRD-Nrg1*^*–/–*^mice, motor neuron numbers were markedly reduced in the spinal cord [38]. To determine if motor neurons were also rescued in *CRD-Nrg1*^*–/–*^*Chat*^*−/−*^mice, we examined cervical segments of spinal cords using anti-choline transporter (CHT) antibodies, which label motor neurons [62]. We found that the somata of motor neurons in the ventral horn of the spinal cord were labeled CHT-positive in both control (S2A Fig, left panels) and *CRD-Nrg1*^*–/–*^*Chat*^*−/−*^mice (S2A Fig, right panels). Furthermore, we counted the numbers of motor axons from phrenic nerves (S2B Fig) and found that the average motor axon numbers per phrenic nerve were similar between control (248 ± 8, N = 3 mice) and *CRD-Nrg1*^*–/–*^*Chat*^*−/−*^(244 ± 11, N = 3 mice) (S2C Fig). Together, these results demonstrated that the loss of phrenic nerve innervation and neuromuscular synapses in single mutant mice deficient in CRD-NRG1 (*CRD-Nrg1*^*–/–*^) were rescued in double mutant mice deficient in both CRD-NRG1 and ChAT (*CRD-Nrg1*^*–/–*^*Chat*^*–/–*^).

### Absence of Schwann cells in *CRD-Nrg1*^*–/–*^*Chat*^*−/−*^mice

**Fig 3:**
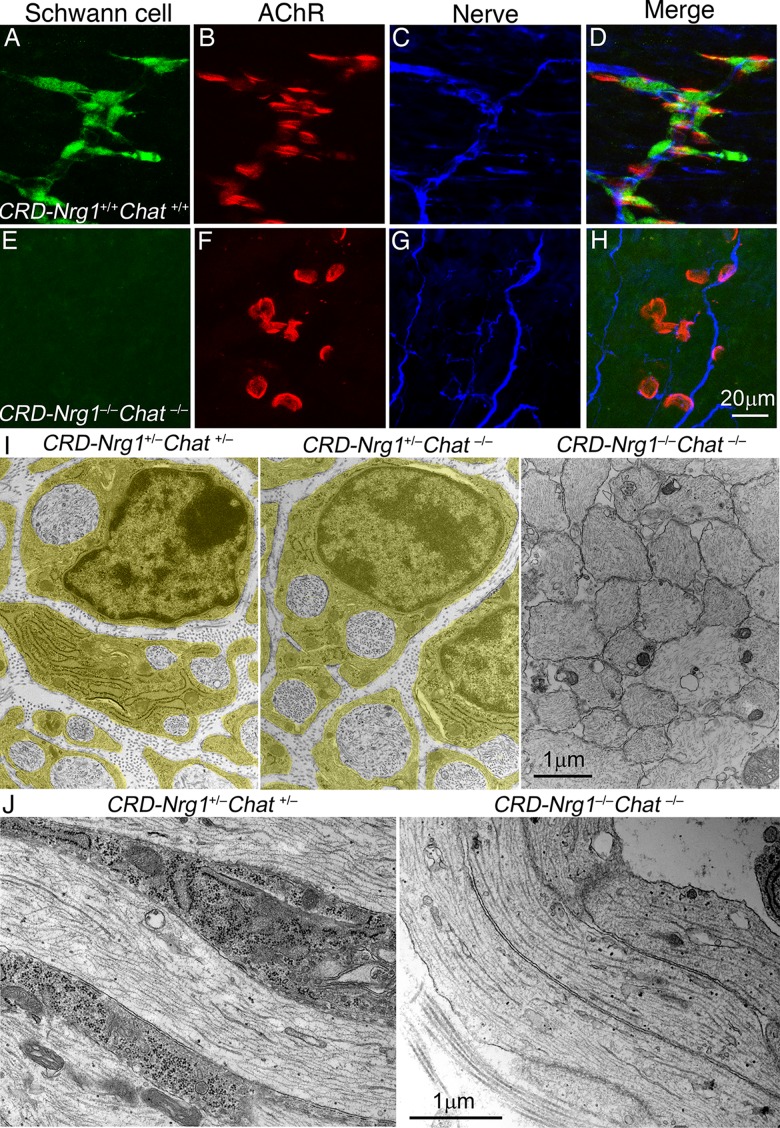
Schwann cells are absent in *CRD-Nrg1*^*–/–*^*Chat*^*−/−*^mice (E18.5). **A-H**: Whole-mounts of diaphragm muscles were triple-labelled with Alexa 647-conjugated α-bungarotoxin (AChR, red), anti-S100β (Schwann cells, green) and anti-neurofilament protein (SMI32, nerve, blue). Schwann cells delineate the nerves in the control (*CRD-Nrg1*^*+/+*^*Chat*^*+/+*^) but are absent in the mutant mice (*CRD-Nrg1*^*–/–*^*Chat*^*–/–*^). Nerves are defasciculated in *CRD-Nrg1*^*–/–*^*Chat*^*–/–*^mice. **I**: cross sections of phrenic nerves from *CRD-Nrg1*^*+/–*^*Chat* ^+/–^, *CRD-Nrg1*^*+/–*^*Chat^−^*^/–^and *CRD-Nrg1*^*–/–*^*Chat^−^*^/–^mice. Schwann cells (pseudo-colored) wrap around individual axons in *CRD-Nrg1*^*+/–*^*Chat* ^+/–^and *CRD-Nrg1*^*+/–*^*Chat^−^*^/–^nerves but are absent in *CRD-Nrg1*^*–/–*^*Chat^−^*^/–^nerves. **J**: Longitudinal sections of the phrenic nerve. Schwann cells processes are present in *CRD-Nrg1*^*+/–*^*Chat* ^+/–^but are absent in *CRD-Nrg1*^*–/–*^*Chat^−^*^/–^mice. Scale bars: A-H: 20μm; I, J: 1 μm.

Using anti-S100β antibodies, which recognize developing Schwann cells [39], we confirmed that Schwann cells were present in control mice but absent in *CRD-Nrg1*^*–/–*^*Chat*^*−/−*^mice (Fig 3E). The absence of Schwann cells was further demonstrated by electron microscopy (EM). In control and Chat single mutant mice (*i*.*e*., *CRD-Nrg1*^*+/–*^*Chat*^*−/−*^mice; Fig 3I and 3J), individual axons were regularly interspersed, and each axon was wrapped by Schwann cell processes. In contrast, phrenic nerves were completely devoid of Schwann cells in *CRD-Nrg1*^*–/–*^*Chat*^*−/−*^mice (Fig 3I and 3J, right panels), and the axons were tightly packed together with very little extracellular space in between.

**Fig 4:**
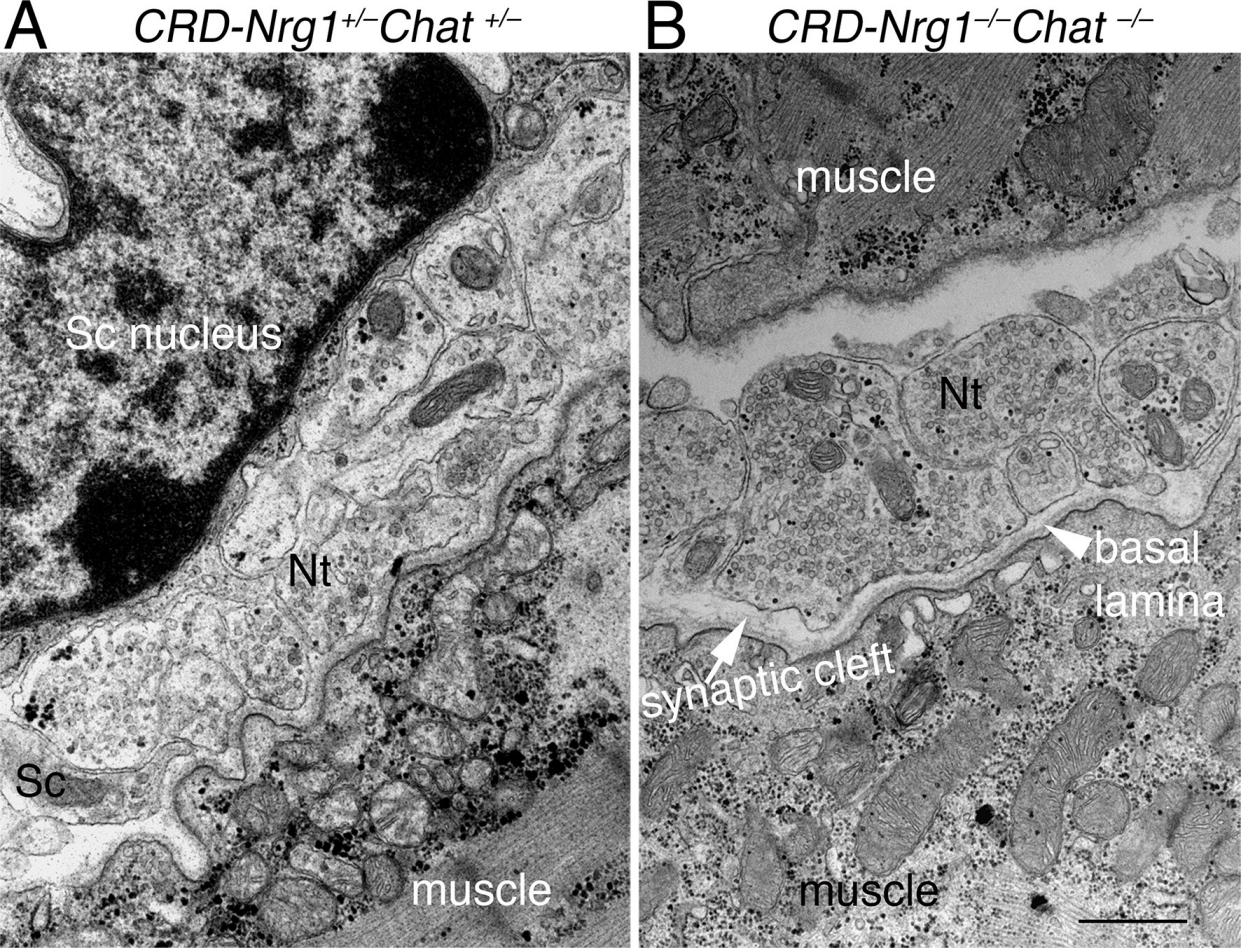
Ultrastructure of “glia-less” synapse in *CRD-Nrg1*^*–/–*^*Chat*^*−/−*^mice (E18.5). Ultrastructure of the NMJs in control (*CRD-Nrg1*^*+/–*^*Chat* ^+/–^, A) and *CRD-Nrg1*^*–/–*^*Chat^−^*^/–^(B) muscles. At the NMJ in *CRD-Nrg1*^*+/–*^*Chat* ^+/–^muscle (A), the nerve terminals are capped by a terminal Schwann cell (Sc). In contrast, the terminal Schwann cell is absent in *CRD-Nrg1*^*–/–*^*Chat^−^*^/–^muscle (B). Despite the lack of the terminal Schwann cell, however, the ultrastructure of the presynaptic nerve terminal, basal lamina and postsynaptic membrane appears similar between control (A) and *CRD-Nrg1*^*–/–*^*Chat^−^*^/–^(B) muscles. Presynaptic nerve terminals contain an abundance of synaptic vesicles and mitochondria, and are separated by a basal lamina in the synaptic cleft. Scale bar: A-B: 1 μm.

Ultrastructural analyses of the NMJs also showed that terminal Schwann cells were absent at the NMJs in *CRD-Nrg1*^*–/–*^*Chat*^*−/−*^mice. Instead, the flanks of presynaptic nerve terminals were directly exposed to the interstitial space in double mutant mice (Fig 4B). No other cell type was found as a substitute for Schwann cells. Occasionally, thin processes of fibroblast-like cells were seen in the interstitial space around the NMJs, but this feature was readily observed in both control and *CRD-Nrg1*^*–/–*^*Chat^−^*^/–^double mutant mice. Although these processes could be seen near the nerve terminal membrane in a few instances, in no case did they wrap, cap or flank the nerve terminals. Consistent with our results in light microscopy (Fig 2D and 2E), no NMJs were observed under EM in E18.5 diaphragm muscles in *CRD-Nrg1*^−/−^mice. In contrast, numerous NMJs were identified under EM in E18.5 diaphragm muscles in control (n = 26), *Chat*^−/−^(n = 14) and *CRD-Nrg1*^−/−^*Chat*^−/−^(n = 18) mice. Among these NMJs, a striking feature was that the numbers of nerve terminal profiles per NMJ were markedly increased in both *Chat*^−/−^and *CRD-Nrg1*^−/−^*Chat*^−/−^mice compared with control mice. Specifically, the numbers of nerve terminal profiles per NMJ were significantly increased in *Chat*^−/−^(10.57 ± 1.75, n = 14, P = 0.004) and *CRD-Nrg1*^−/−^*Chat*^−/−^(15.28 ± 1.86, n = 18, P = 4.707 x 10^−6^), respectively, compared with the control (6.17 ± 0.53, n = 26). The difference in presynaptic nerve terminal numbers per NMJ between *Chat*^−/−^and *CRD-Nrg1*^−/−^*Chat*^−/−^mice, however, was not statistically significant (P = 0.083).

Strikingly, despite the absence of terminal Schwann cells, the ultrastructure of presynaptic nerve terminals in *CRD-Nrg1*^−/−^*Chat*^−/−^mice appeared similar to that of control mice: multiple nerve terminals, each with abundant synaptic vesicles, mitochondria, glycogen granules, and other membranous structures, were observed. The postsynaptic compartment also displayed typical ultrastructural features: electro-dense postsynaptic membranes and abundant sub-synaptic organelles, such as mitochondria and ribosomes (compare Fig 4A to Fig 4B). Thus, bipartite neuromuscular synapses composed of only presynaptic nerve terminals and postsynaptic muscle membrane were established in the absence of Schwann cells in *CRD-Nrg1*^*–/–*^*Chat^−^*^/–^mice.

### Ablating post-synaptic AChRs preserves NMJs in *CRD-Nrg1*^*–/–*^mice

AChRs are expressed at multiple sites within the NMJ, including presynaptic nerve terminals, terminal Schwann cells and postsynaptic muscles [10, 63]. Although Schwann cells were absent in *CRD-Nrg1*^*–/–*^mice, it remained possible that a blockade of cholinergic transmission (as in *CRD-Nrg1*^*–/–*^*Chat^−^*^/–^mice) may affect synaptic transmission at either pre-synaptic or post-synaptic sites, or both. To determine whether the effects of blocking cholinergic synaptic transmission were mediated through postsynaptic AChRs, we examined the NMJs of mice selectively lacking postsynaptic AChRs–mice lacking the gene encoding the AChR α1 subunit (*Chrna1*^*–/–*^mutants), which is selectively expressed in muscle but not in motor neurons or Schwann cells [64].

**Fig 5:**
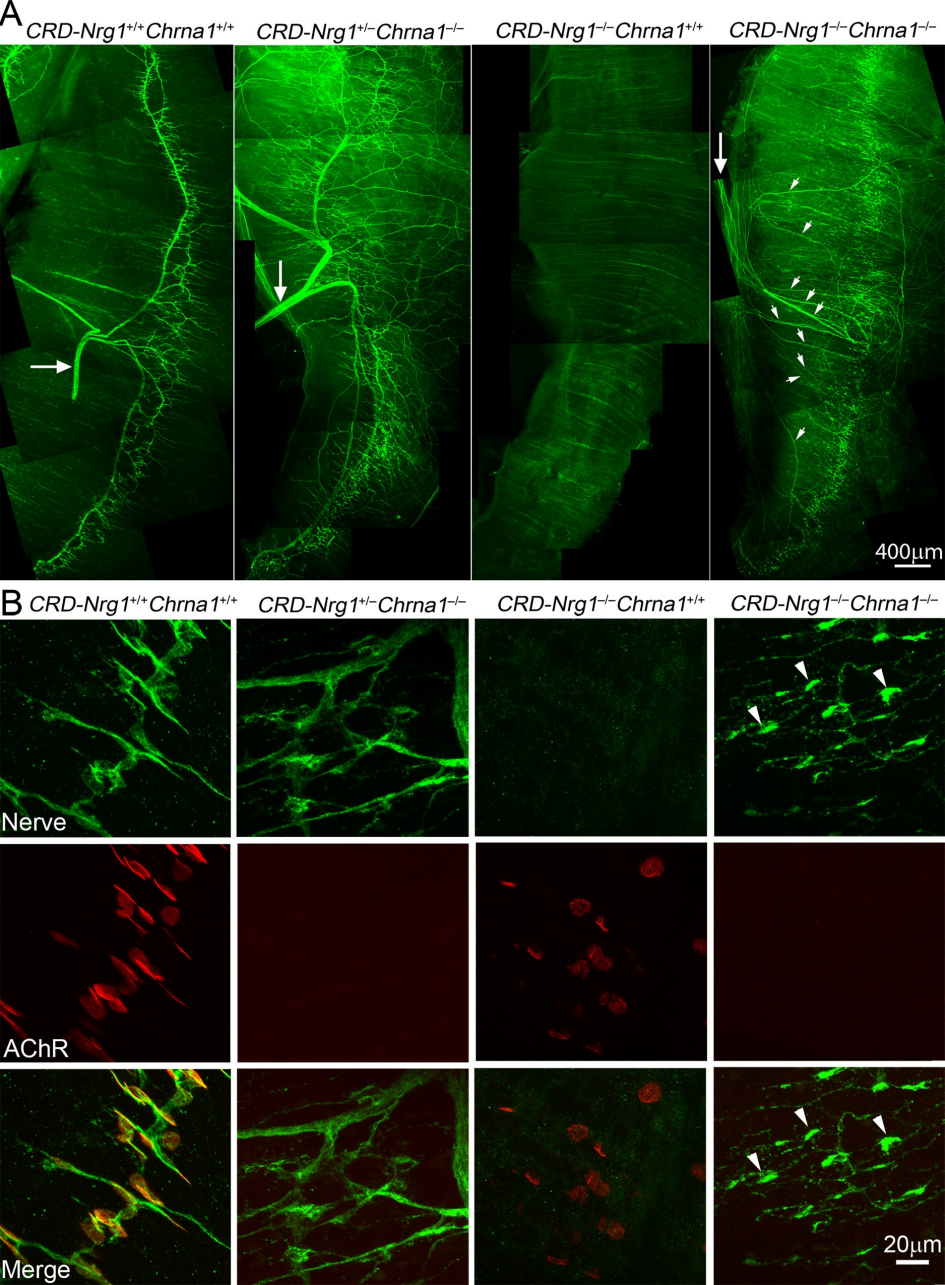
Ablating postsynaptic AChRs (*Chrna1*^*–/–*^) preserves innervation in *CRD-Nrg1*^*–/–*^*Chrna1*^*–/–*^mice. **A**: Low power views of hemi diaphragm muscle (E18.5) from wild-type (CRD-Nrg1^+/+^Chrna1^+/+^), *Chrna1*^*–/–*^, *CRD-Nrg1*^*–/–*^*and CRD-Nrg1*^*–/–*^*Chrna1*^*–/–*^mice, labeled with anti-syntaxin1 antibodies (green), illustrating innervation patterns (large arrows point to phrenic nerve trunks): the nerves are absent in *CRD-Nrg1*^*–/–*^*Chrna1*^*+/+*^ muscle but are established in *CRD-Nrg1*^*–/–*^*Chrna1*^*–/–*^muscle (small arrowheads point to defasciculated intramuscular nerves). **B**: High power images of embryonic diaphragm muscles (E18.5) were labeled by anti-syntaxin1 antibodies (green) and Texas Red conjugated α-bgt (red). AChRs are absent in *CRD-Nrg1*^*+/–*^*Chrna1*^*–/–*^and *CRD-Nrg1*^*–/–*^*Chrna1*^*–/–*^muscles due to *Chrna1* deficiency. CRD-Nrg1 deficiency led to an absence of innervation in *CRD-Nrg1*^*–/–*^*Chrna1*^*+/+*^ muscles. Deleting both CRD-Nrg1 and Chrna1 (*CRD-Nrg1*^*–/–*^*Chrna1*^*–/–*^) restored innervation, and the nerve terminals in *CRD-Nrg1*^*–/–*^*Chrna1*^*–/–*^muscles are intensely labeled by anti-syntaxin1 antibodies (arrowheads). Scale bars: A: 400 μm; B: 20 μm.

We found that muscle innervation was also completely rescued in double mutant mice lacking both AChRα1 and CRD-Nrg1 (*CRD-Nrg1*^*–/–*^*Chrna1*^*–/–*^) (Fig 5A), similar to the results displayed in *CRD-Nrg1*^*–/–*^*Chat*^*–/–*^(Fig 2D) and *CRD-Nrg1*^*–/–*^*Snap25*^*–/–*^mice (Fig 1A). Furthermore, the rescued motor nerve terminals in *CRD-Nrg1*^*–/–*^*Chrna1*^*–/–*^mice were intensely labeled by anti-syntaxin1 antibodies (Fig 5B), indicating that pre-synaptic terminal differentiation proceeded in the absence of AChRs, which is consistent with previous results shown in *Chrna1*^*–/–*^mice [64].

### Deleting muscle DHPRs preserves NMJs in the absence of Schwann cells

Muscle AChRs are nonselective cation channels, and their activation leads to an influx of cations, including Na^+^ and Ca^2+^, and triggers muscle action potentials (muscle electrical activity), mediated by the voltage-gated sodium channels. Muscle action potentials depolarize the sarcolemma and activate voltage-sensitive dihydropyridine receptors (DHPRs), the L-type Ca^2+^ channels localized to the T tubules, and ultimately activate ryanodine receptors (RyR) in the sarcoplasmic reticulum [65, 66]. It has been previously shown that genetic deletion of *Cacnb1*, the gene encoding the β1 subunit of DHPRs, blocks DHPR function [67] without affecting muscle electrical activity [68].

**Fig 6:**
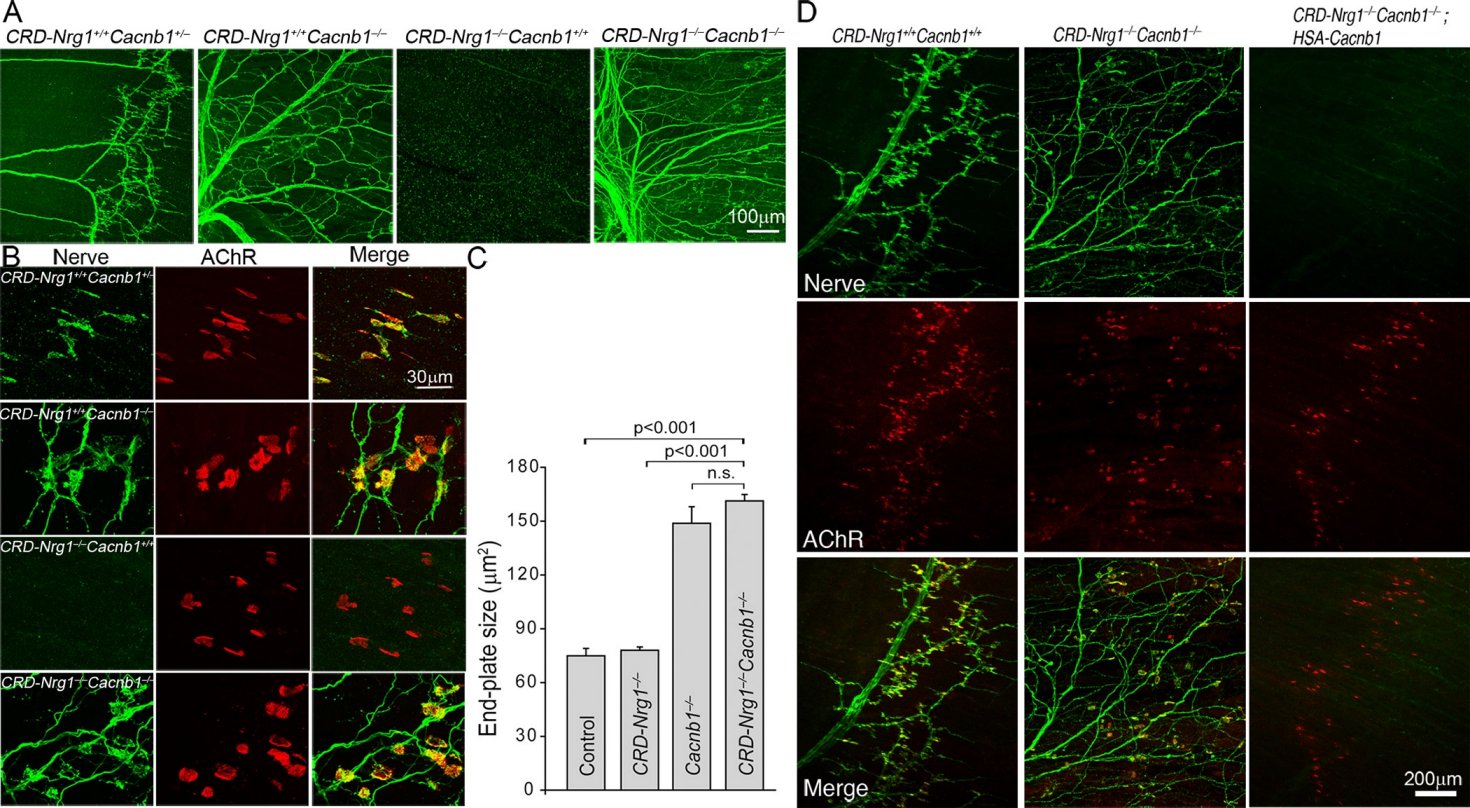
Removal of skeletal muscle dihydropyridine receptors (*Cacnb1*^*–/–*^) prevents synapse loss in *CRD-Nrg1*^*–/–*^mice. Immunofluorescence staining of embryonic diaphragm muscles (E18.5) using anti-NF150 and anti-syt2 antibodies (green), and Texas Red-conjugated α-bgt (red). **A:** Innervation patterns in the indicated genotypes. Muscles from *CRD-Nrg1*^*–/–*^*Cacnb1*^*+/+*^ mice lack innervation, and this phenotype is rescued in *CRD-Nrg1*^*–/–*^*Cacnb1*^*–/–*^muscles, despite the nerve being highly defasciculated (arrow). **B:** High-power images of individual NMJs. The nerve terminals are absent in *CRD-Nrg1*^*–/–*^*Cacnb1*^*+/+*^ muscles. In contrast, the nerve terminals were juxtaposed with AChR clusters in *CRD-Nrg1*^*–/–*^*Cacnb1*^*–/–*^muscles. **C**: Quantitative analyses of postsynaptic endplate size. The average endplate size is similar between control and *CRD-Nrg1*^−/−^mice: 74.9 ± 4.1 μm^2^ in control (N = 3, n = 287) and 78.0 ± 1.8 μm^2^ (N = 3, n = 239) in *CRD-Nrg1*^−/−^mice. In contrast, the average endplate size is increased 2-fold to 148.8 ± 9.2 μm^2^ in *Cacnb1*^−/−^mice (N = 3, n = 243) and to 161.3 ± 3.6 μm^2^ in *CRD-Nrg1*^−/−^*Cacnb1*^−/−^mice (N = 3, n = 233), compared with control. N: number of mice; n: number of end-plates. **D**: Innervation and AChR patterns in the diaphragm muscles from *CRD-Nrg1*^*+/+*^*Cacnb1*^*+/+*^, *CRD-Nrg1*^*–/–*^*Cacnb1*^*–/–*^and *CRD-Nrg1*^*–/–*^*Cacnb1*^*–/–*^expressing *HSA-Cacnb1* transgene (*CRD-Nrg1*^*–/–*^*Cacnb1*^*–/–*^; *HSA-Cacnb1*). Transgenic expression of Cacnb1 in skeletal muscle (*HSA-Cacnb1*) (*CRD-Nrg1*^−/−^*Cacnb1*^−/−^; *HSA-Cacnb1*) prevents the rescuing of muscle innervation and synapse loss in *CRD-Nrg1*^−/−^*Cacnb1*^−/−^mice. Scale bars: A: 100 μm; B: 30 μm; D: 200 μm.

To determine the role of muscle activity mediated by DHPRs in NMJ formation in the absence of Schwann cells (*i*.*e*., in *CRD-Nrg1*^−/−^mice), we examined double mutant mice deficient in both *CRD-Nrg1* and *Cacnb1*. Remarkably, ablating skeletal muscle DHPRs (*Cacnb1*^*–/–*^) in *CRD-Nrg1*^*–/–*^mice (*i*.*e*., in *CRD-Nrg1*^*–/–*^*Cacnb1*^*–/–*^) prevented muscle denervation (Fig 6A). And, similar to those observed in *CRD-Nrg1*^*–/–*^*Snap25 ^–^*^*/–*^(Fig 1A), *CRD-Nrg1*^*–/–*^*Chat*^*–/–*^(Fig 2C and 2D, S1 Fig), and *CRD-Nrg1*^*–/–*^*Chrna1*^*–/–*^(Fig 5A) mice, intramuscular nerves in *CRD-Nrg1*^*–/–*^*Cacnb1*^*–/–*^were highly defasciculated (Fig 6A). Furthermore, neuromuscular synapses were established in *CRD-Nrg1*^*–/–*^*Cacnb1*^*–/–*^mice. We counted 233 end-plates from E18.5 diaphragm muscles in *CRD-Nrg1*^*–/–*^*Cacnb1*^*–/–*^mice (N = 3 mice), and found that 100% of AChR clusters were juxtaposed to the pre-synaptic nerve terminals (Fig 6B). Furthermore, the average size of the postsynaptic endplate increased more than 2-fold in *CRD-Nrg1*^−/−^*Cacnb1*^−/−^mice (161.3 ± 3.6 μm^2^, N = 3, n = 233), compared with control (74.9 ± 4.1 μm^2^, N = 3, n = 287) or *CRD-Nrg1*^−/−^mice (78.0 ± 1.8 μm^2^, N = 3, n = 239) (Fig 6C). Importantly, re-introducing Cacnb1 back into skeletal muscle by transgenic expression of Cacnb1 using a muscle-specific promoter–human skeletal muscle actin (*HSA-Cacnb1*) in *CRD-Nrg1*^−/−^*Cacnb1*^−/−^mice–abolished the rescue of muscle innervation and synapse loss (Fig 6D). Therefore, ablating Cacnb1 in skeletal muscles is specifically required for the rescue of muscle innervation and synapse loss in *CRD-Nrg1*^−/−^*Cacnb1*^−/−^mice.

**Fig 7:**
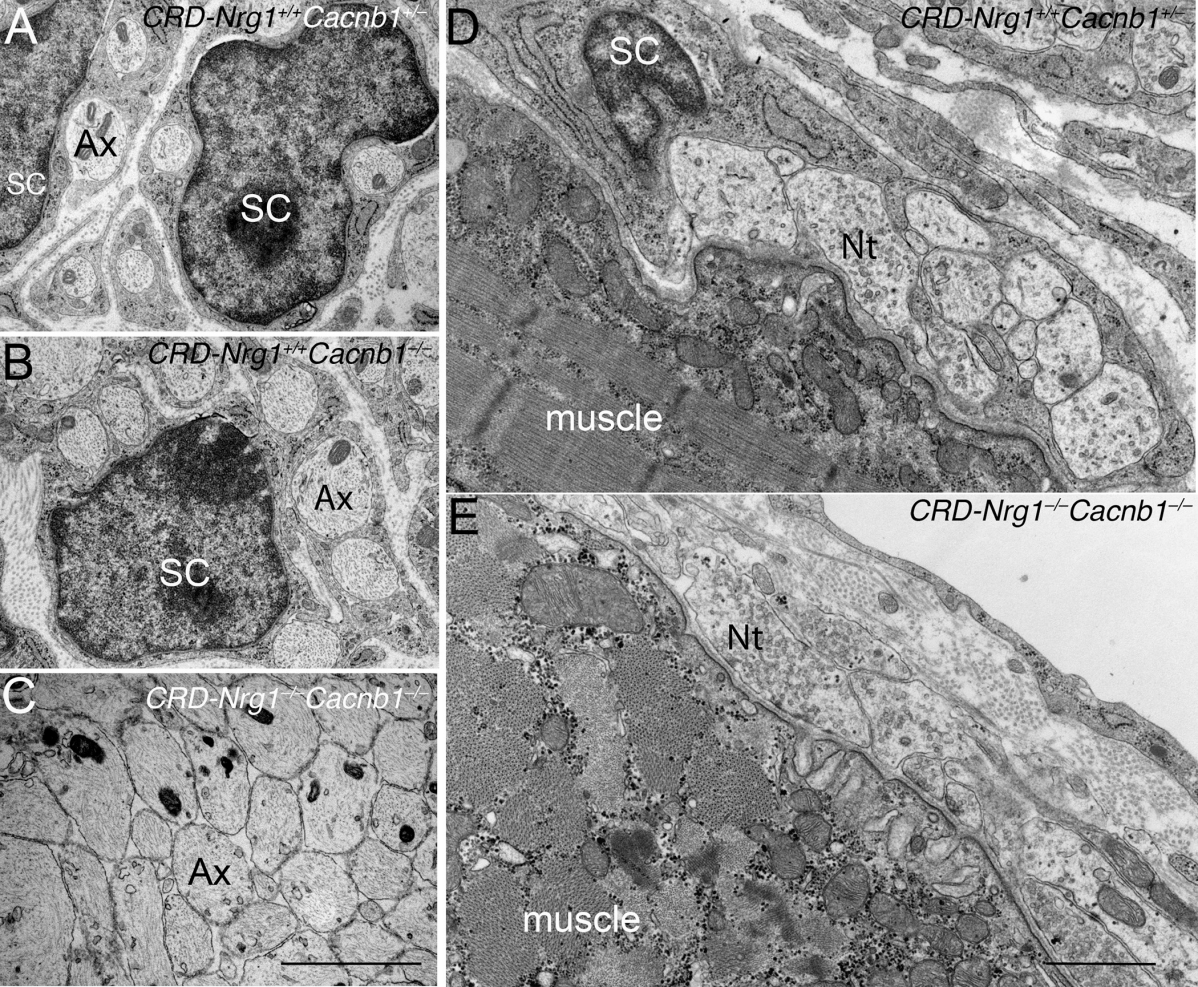
Ultrastructural analyses of the nerves and NMJs in *CRD-Nrg1*^*–/–*^*Cacnb1 –*^*/–*^mice (E18.5). **A-C**: Phrenic nerve cross sections from *CRD-Nrg1*^*+/+*^*Cacnb1* ^+/–^, *CRD-Nrg1*^*+/+*^*Cacnb1 ^–^*^/–^and *CRD-Nrg1*^*–/–*^*Cacnb1 –*^/–^mice. Schwann cells (SC) are absent in *CRD-Nrg1*^*–/–*^*Cacnb1 ^–^*^/–^nerves. **D-E**: Ultrastructure of the NMJs in control (*CRD-Nrg1*^*+/+*^*Cacnb1* ^+/–^(D) and *CRD-Nrg1*^*–/–*^*Cacnb1 ^–^*^/–^(E) muscles. A terminal Schwann cell caps the nerve terminals (Nt) in *CRD-Nrg1*^*+/+*^*Cacnb1* ^+/–^(D) but is absent in *CRD-Nrg1*^*–/–*^*Cacnb1 ^–^*^/–^muscle (E). Despite the lack of terminal Schwann cells, the ultrastructure of the presynaptic nerve terminal, basal lamina and postsynaptic membrane appear similar between control (D) and *CRD-Nrg1*^*–/–*^*Cacnb1 ^–^*^/–^muscles (E). Scale bar: A-C: 2 μm; D-E: 1 μm.

Ultrastructural analysis confirmed that Schwann cells were indeed absent in the nerves (Fig 7C) and the NMJs in *CRD-Nrg1*^*–/–*^*Cacnb1*^−/−^mice (Fig 7E). Similar to the results obtained with the NMJs in *CRD-Nrg1*^*–/–*^*Chat*^*−/−*^mice, the numbers of pre-synaptic nerve terminals per NMJ were markedly increased in *CRD-Nrg1*^*–/–*^*Cacnb1*^−/−^mice, compared with controls. On average, there were 9.44 ± 1.03 (n = 26) nerve terminals at the NMJs in *CRD-Nrg1*^*–/–*^*Cacnb1*^−/−^, 8.68 ± 0.93 (n = 19) in *Cacnb1*^−/−^, and 6.29 ± 0.70 (n = 21) in control mice. While the nerve terminal numbers were not statistically different between *CRD-Nrg1*^*–/–*^*Cacnb1*^−/−^and *Cacnb1*^−/−^mice, the nerve terminal numbers were significantly (P = 0.019) increased in *CRD-Nrg1*^*–/–*^*Cacnb1*^−/−^mice, compared with those observed in control mice. However, the ultrastructure of the presynaptic nerve terminal, basal lamina and postsynaptic membrane appeared remarkably similar to those in control NMJs (compare Fig 7D to Fig 7E), except that terminal Schwann cells were absent in *CRD-Nrg1*^*–/–*^*Cacnb1*^−/−^mice (Fig 7E).

### Deleting muscle Ryr1 preserves NMJs in the absence of schwann cells

**Fig 8:**
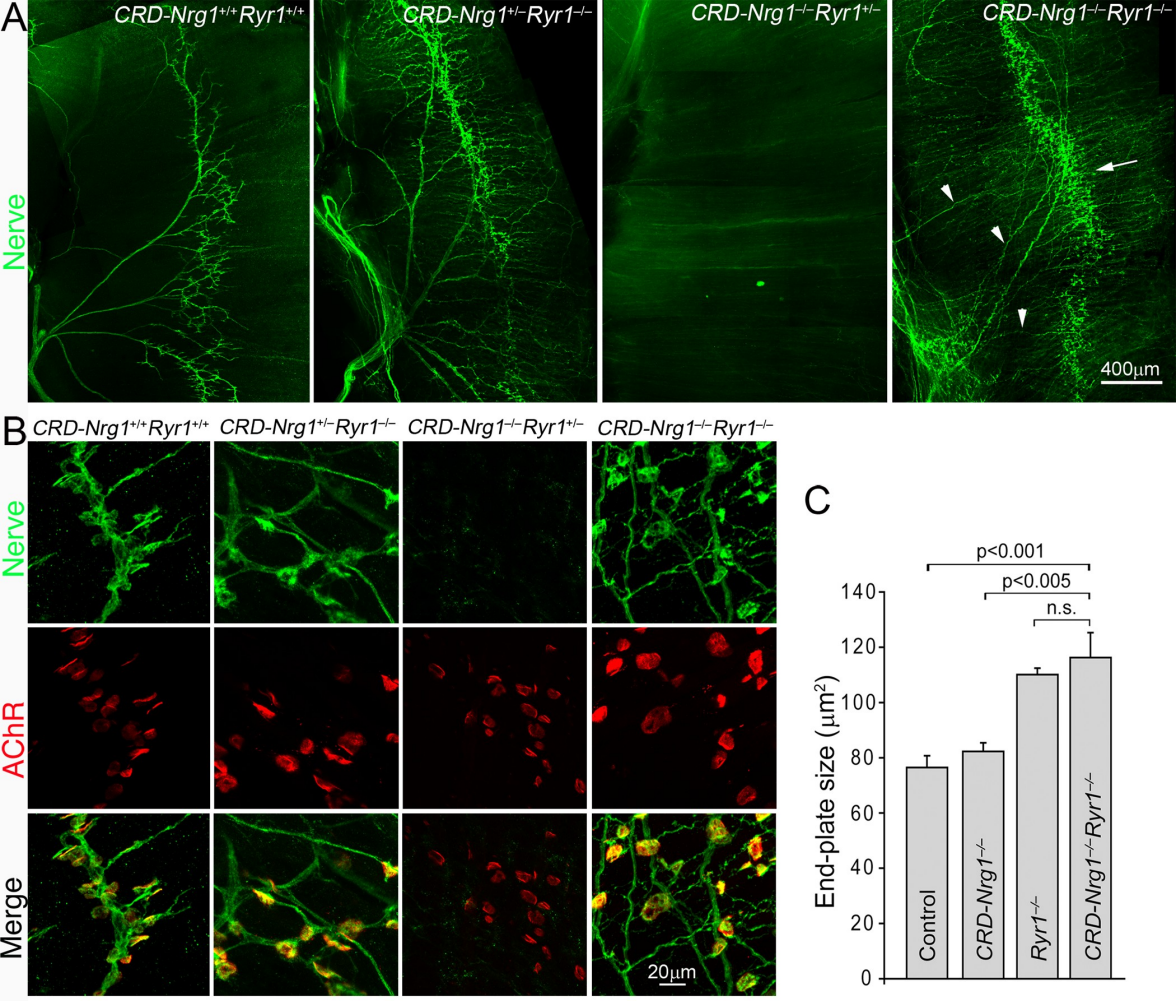
Ablating muscle ryanodine receptor 1 (*Ryr1*^*–/–*^) rescues denervation and preserves NMJs in *CRD-Nrg1*^*–/–*^mice. Embryonic diaphragm muscles (E18.5) were double-labeled by anti-syntaxin 1 antibodies to reveal innervation pattern (green), and by Texas Red-conjugated α-bgt to visualize endplates (red). **A**: Low-power views of the innervation patterns in the dorsal quadrants of diaphragm muscles from the indicated genotypes. **B**: High-power images of individual NMJs. The muscle fibers in *CRD-Nrg1*^*–/–*^*Ryr1*^*+/+*^ mice were completely denervated (A), and their endplates were vacant (B). These defects were rescued with robust innervation and NMJs were established, in *CRD-Nrg1*^*–/–*^*Ryr1*^*–/–*^muscle. Arrowheads in A point to defasciculation of the intramuscular nerves, and the arrow points to the nerve terminals localized along the central region in *CRD-Nrg1*^*–/–*^*Ryr1*^*–/–*^muscle. **C:** Quantification of postsynaptic endplate size. The endplate size is significantly increased in *Ryr1*^−/−^(110.1 ± 2.4 μm^2^, N = 3, n = 266) and *CRD-Nrg1*^−/−^*Ryr1*^−/−^(116.3 ± 9.0 μm^2^, N = 3, n = 242) mice, compared with control (76.5 ± 4.3 μm^2^, N = 3, n = 208) or *CRD-Nrg1*^−/−^(82.3 ± 3.1 μm^2^, N = 3, n = 230). N: number of mice; n: number of endplates. Scale bars: A: 400 μm; B: 20 μm.

The activation of muscle DHPRs leads to the activation of muscle ryanodine receptors (RyRs), triggering the release of Ca^2+^ from the sarcoplasmic reticulum into the muscle cytosol, leading to muscle contractions, a process generally referred to as excitation-contraction coupling (E-C coupling) [65, 69–72]. There are three Ryr genes in rodents—Ryr1, Ryr2 and Ryr3, each with distinct expression patterns: Ryr1 is predominantly expressed in skeletal muscle, Ryr2 in cardiac muscle and brain, and Ryr3 in brain [73–80]. To determine if muscle activity mediated by ryanodine receptors plays a role in NMJ formation in *CRD-Nrg1*^*–/–*^mice, we examined double mutant mice deficient in both CRD-Nrg1 and Ryr1 (*CRD-Nrg1*^*–/–*^*Ryr1*^*–/–*^). We found that, similar to *CRD-Nrg1*^*–/–*^*Cacnb1*^*–/–*^mice, neuromuscular synapse loss and muscle denervation were rescued in *CRD-Nrg1*^*–/–*^*Ryr1*^*–/–*^mice (Fig 8A and 8B). Specifically, the diaphragm muscles in *CRD-Nrg1*^*–/–*^*Ryr1*^*–/–*^mice were robustly innervated by highly defasciculated nerves (Fig 8A), similar to those observed in *CRD-Nrg1*^*–/–*^*Snap25*^*–/–*^(Fig 1A), *CRD-Nrg1*^*–/–*^*Chat−*^/–^(Fig 2C and 2D, S1 Fig), *CRD-Nrg1*^*–/–*^*Chrna1*^*–/–*^(Fig 5A and 5B) and *CRD-Nrg1*^*–/–*^*Cacnb1*^*–/–*^(Fig 6A) mice. Pre-synaptic nerve terminals were juxtaposed with post-synaptic AChR clusters in *CRD-Nrg1*^*–/–*^*Ryr1*^*–/–*^mice (Fig 8B). Furthermore, the average size of postsynaptic endplates was significantly increased in *CRD-Nrg1*^−/−^*Ryr1*^−/−^mice, compared with those of control mice (Fig 8C). Together, these results demonstrate that blocking E-C coupling, similar to blocking muscle excitation (as in *CRD-Nrg1*^*–/–*^*Cacnb1*^−/−^mice), is sufficient to reverse the loss of neuromuscular synapses in *CRD-Nrg1*^*–/–*^mice.

### Schwann cell-deficient neuromuscular synapses exhibited increased spontaneous synaptic activity

**Fig 9:**
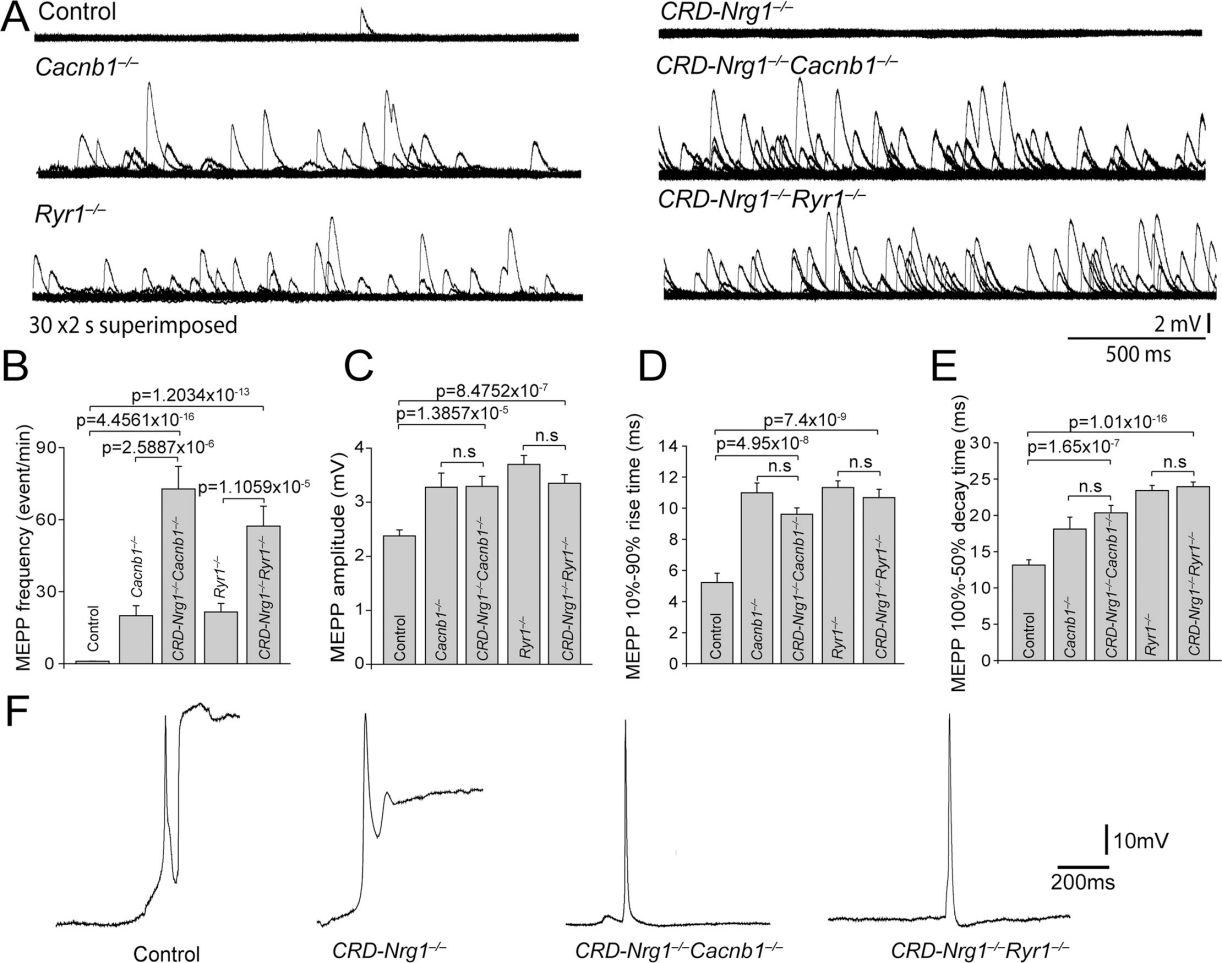
The absence of Schwann cells leads to increased spontaneous synaptic activity. **A**: Sample traces of mEPPs recorded in diaphragm muscles from control, *CRD-Nrg1*^−/−^, *Cacnb1*^−/−^, *Ryr1*^−/−^, *CRD-Nrg1*^−/−^*Cacnb1*^−/−^and *CRD-Nrg1*^−/−^*Ryr1*^−/−^mice (E18.5). Each trace in A represents a 1-minute, continuous recording (30 superimposed 2-second sweeps). MEPP (arrowhead, A) typically occurred at a low frequency (average at 1 event per minute) in control mice. No mEPPs were detected in *CRD-Nrg1*^−/−^mice (N = 6 mice, n = 36 cells). In contrast, markedly increased mEPP frequencies were detected in *Cacnb1*^−/−^, *Ryr1*^−/−^, *CRD-Nrg1*^−/−^*Cacnb1*^−/−^and *CRD-Nrg1*^−/−^*Ryr1*^−/−^muscles. **B-E**: Quantitative analyses of mEPP frequencies (events/min) (**B**), amplitudes (mV) (**C**), rise time (10–90% rise time) (**D**) and decay time (half-decay time, 100–50%) (**E**). Sample size: control: N = 8 mice, n = 65 cells; *CRD-Nrg1*^−/−^: N = 6 mice, n = 36 cells; *CRD-Nrg1*^−/−^*Cacnb1*^−/−^: N = 3 mice, n = 42 cells; *Cacnb1*^−/−^: N = 3 mice, n = 39 cells; *CRD-Nrg1*^−/−^*Ryr1*^−/−^: N = 3 mice, n = 41 cells; *Ryr1*^−/−^: N = 7 mice, n = 75 cells. **F**: Spontaneous muscle action potentials. Arrows in F point to traces caused by the displacement of recording electrodes resulted from muscle contractions. No such displacements were ever recorded in *CRD-Nrg1*^−/−^*Cacnb1*^−/−^and *CRD-Nrg1*^−/−^*Ryr1*^−/−^muscles, as these muscles failed to contract.

Presynaptic nerve terminals at the NMJ release neurotransmitters spontaneously, leading to miniature end plate potentials (mEPPs) in post-synaptic muscle fibers [81, 82]. To determine the levels of spontaneous synaptic activity at the NMJs lacking Schwann cells, we carried out electrophysiological analyses in acutely isolated diaphragm muscle/phrenic nerve preparations. No spontaneous synaptic activity was detected in *CRD-Nrg1*^−/−^muscles (N = 6 mice, n = 36 cells), since the diaphragm muscles in *CRD-Nrg1*^−/−^muscles were completely denervated due to withdrawal of the nerve terminals. However, spontaneous muscle action potentials were readily observed in *CRD-Nrg1*^−/−^muscles–similar to spontaneous muscle action potentials recorded in control mice–and muscle contraction was observed following spontaneous muscle action potentials (Fig 9F). Spontaneous muscle action potentials were also observed in *CRD-Nrg1*^−/−^*Cacnb1*^−/−^and *CRD-Nrg1*^−/−^*Ryr1*^−/−^muscles, but these mutant muscles failed to contract after firing action potentials (Fig 9F).

Notably, significantly increased spontaneous synaptic activity was detected in both *CRD-Nrg1*^−/−^*Cacnb1*^−/−^and *CRD-Nrg1*^−/−^*Ryr1*^−/−^double mutant mice, compared with that in control, *Cacnb1*^−/−^, or *Ryr1*^−/−^single mutant mice (Fig 9A). In control mice, mEPP frequencies were approximately 1 event per minute (1.0 ± 0.1, n = 65 cells, N = 8 mice) (Fig 9B). Strikingly, mEPP frequencies increased 7200% over control in *CRD-Nrg1*^−/−^*Cacnb1*^−/−^mice (72.8 ± 9.4, n = 42 cells, N = 3 mice). Furthermore, mEPP frequencies in *CRD-Nrg1*^−/−^*Cacnb1*^−/−^mice were 260% higher than that in *Cacnb1*^−/−^mice (20.1 ± 4.1, n = 39 cells, N = 3 mice) (Fig 9B). Similarly, mEPP frequencies in *CRD-Nrg1*^−/−^*Ryr1*^−/−^mice (57.3 ± 8.3, n = 41 cells, N = 3 mice) were also significantly increased, compared with those in *Ryr1*^−/−^(21.6 ± 3.5, n = 75 cells, N = 7 mice) (Fig 9B).

MEPP amplitudes (Fig 9C), rise time (10–90%) (Fig 9D) and half-decay time (Fig 9E) were significantly increased in both *CRD-Nrg1*^−/−^*Cacnb1*^−/−^(amplitude: 3.29 ± 0.19 mV, rise time: 9.61 ± 0.41 ms; half-decay time: 20.35 ± 1.00 ms) and *CRD-Nrg1*^−/−^*Ryr1*^−/−^(amplitude: 3.35 ± 0.16 mV, rise time: 10.68 ± 0.54 ms; half-decay time: 23.94 ± 0.65 ms) mice, compared with control (amplitude: 2.38 ± 0.11 mV, rise time: 5.23 ± 0.60 ms; half-decay time: 13.16 ± 0.71 ms). However, MEPP amplitudes, rise time (10–90%) and half-decay time were not significantly different between *CRD-Nrg1*^−/−^*Cacnb1*^−/−^and *Cacnb1*^−/−^mice (amplitude: 3.28 ± 0.26 mV, rise time: 10.99 ± 0.63 ms; half-decay time: 18.12 ± 1.64 ms), or between *CRD-Nrg1*^−/−^*Ryr1*^−/−^and *Ryr1*^−/−^mice (amplitude: 3.70 ± 0.17 mV, rise time: 11.33 ± 0.43 ms; half-decay time: 23.41 ± 0.71 ms) (Fig 9C–9E). Thus, the increase in mEPP frequencies in *CRD-Nrg1*^−/−^*Cacnb1*^−/−^and *CRD-Nrg1*^−/−^*Ryr1*^−/−^mice appeared to be the common, and, significant change between NMJs without Schwann cells (i.e., in *CRD-Nrg1*^−/−^*Cacnb1*^−/−^and *CRD-Nrg1*^−/−^*Ryr1*^−/−^mice) and those with Schwann cells (i.e., in *Cacnb1*^−/−^and *Ryr1*^−/−^mice). This suggests that the increase in spontaneous synaptic activity in both *CRD-Nrg1*^−/−^*Cacnb1*^−/−^and *CRD-Nrg1*^−/−^*Ryr1*^−/−^NMJs is attributable to the pre-synaptic elements. Together, these data demonstrated that neuromuscular synapses lacking Schwann cells exhibit increased pre-synaptic activity.

## Discussion

**Fig 10:**
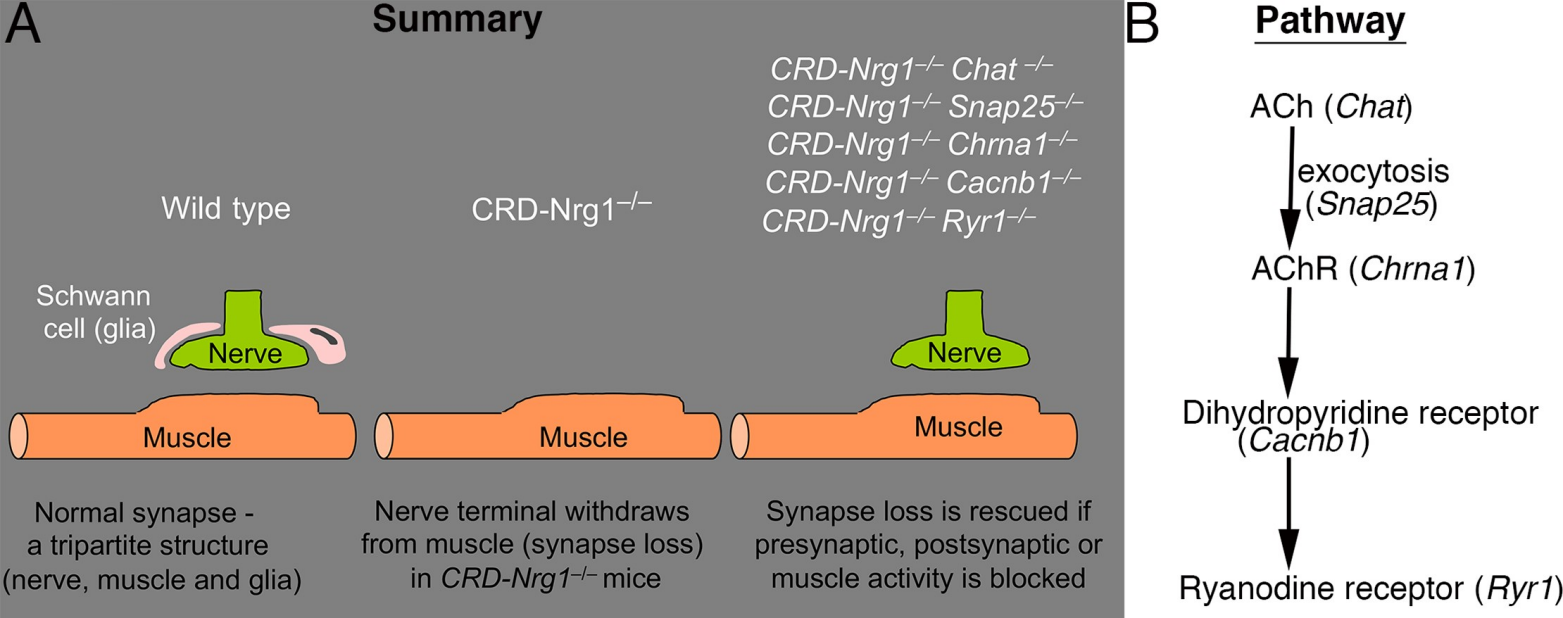
The main findings are illustrated (**A**). Neuromuscular synapses (NMJs) are normally formed as a tripartite structure including a presynaptic nerve terminal (green), a postsynaptic muscle (orange) and a Schwann cell (glia, pink). Schwann cells are essential for the formation of the NMJ; in the absence of Schwann cells, nerve terminals withdraw from muscles, resulting in synapse loss (synapse degeneration) and muscle denervation (as shown in *CRD-Nrg1*^−/−^mice). These defects are rescued by (1) a blockade of evoked neurotransmitter release (*CRD-Nrg1*^−/−^*Snap25*^−/−^); (2) a blockade of neurotransmitter synthesis (*CRD-Nrg1*^−/−^*Chat^−^*^/–^); (3) a blockade of post-synaptic AChRs (*CRD-Nrg1*^−/−^*Chrna1*^−/−^); (4) a blockade of muscle dihydropyridine receptors (*CRD-Nrg1*^−/−^*Cacnb1*^−/−^); and (5) a blockade of muscle ryanodine receptor 1 (*CRD-Nrg1*^−/−^*Ryr1*^−/−^). These genetic rescues reveal a common pathway (**B**) that ultimately leads to muscle activity mediated by DHPR/Ryr1. Therefore, a blockade of muscle activity is the key to rescuing muscle denervation/synapse loss in the absence of Schwann cells. Together, these genetic manipulations indicate that the blockade of muscle activity prevents muscle denervation and neuromuscular synapse loss caused by CRD-NRG1 deficiencies in mice.

Terminal Schwann cells are normally required for the assembly of the classic tripartite structure of the NMJs. Mutant mice deficient in type III Neuregulin 1, as well as mutant mice deficient in erbB2 or erbB3, lack Schwann cells, and consequently, lose the NMJs in the diaphragm muscle, after a transient nerve-muscle contact during development [37–41]. In this study, we found that NMJs can be established in *CRD-Nrg1*^−/−^mice, in the absence of Schwann cells, if either synaptic or muscle activity is blocked. These findings are surprising because it shows that bipartite NMJs can be established *in vivo*, in the absence of Schwann cells. Specifically, neuromuscular synapses are established in the absence of Schwann cells in *CRD-Nrg1*^−/−^mice if one of the following genes is also ablated: *Snap25* (neurotransmitter release), *Chat* (cholinergic transmission), *Chrna1* (postsynaptic AChR), *Cacnb1* (skeletal muscle DHPRs, the voltage sensor and L-type Ca^2+^ channel on the muscle membrane) or *Ryr1* (skeletal muscle ryanodine receptors). These genetic manipulations follow a pathway that ultimately leads to muscle activity mediated by (DHPR/RyR) (Fig 10). Importantly, electrophysiological analyses revealed robust synaptic activity in the rescued, Schwann-cell deficient NMJs in *CRD-Nrg1*^−/−^*Cacnb1*^−/−^or *CRD-Nrg1*^−/−^*Ryr1*^−/−^mutant mice. Thus, a blockade of synaptic activity, although sufficient, is not necessary to preserve NMJs that lack Schwann cells. Instead, a blockade of muscle activity mediated by DHRPs and Ryr1 is both necessary and sufficient for preserving NMJs that lack Schwann cells.

How might muscle inactivity contribute to the formation of neuromuscular synapses in the absence of Schwann cells? We can envision several distinct mechanisms. The first possibility is that muscle inactivity (*i*.*e*., a blockade of muscle activity) leads to increases in nerve branching, as shown previously [60, 61, 64, 83–85], which might accordingly increase the access of Schwann cell-deficient synapses to trophic factors that may preserve synapses [see review in ref. [86]]. If this mechanism were to operative, one might expect to observe an increase in nerve branching and hence trophic access in mutants lacking both activity and Schwann cells. However, our data contradict this scenario because, despite marked increases in nerve defasciculation, no increases in nerve branching were detected in Schwann cell-deficient mutants, compared with mutant mice that retain Schwann cells (*i*.*e*., compare *CRD-Nrg1*^*–/–*^*Snap25 ^–^*^/–^versus *Snap25 ^–^*^/–^; *CRD-Nrg1*^*–/–*^*Chat^−^*^/–^versus *Chat^−^*^/–^; *CRD-Nrg1*^*–/–*^*Chrna1*^−/−^versus *Chrna1*^−/−^; *CRD-Nrg1*^*–/–*^*Cacnb1*^*–/–*^versus *Cacnb1*^*–/–*^; and *CRD-Nrg1*^*–/–*^*Ryr1*^*–/–*^versus *Ryr1*^*–/–*^mice). These data further indicate that the well-documented increases in nerve branching induced by inactivity [60, 61, 64, 83–85] require the presence of Schwann cells.

A second possibility is that, muscle fibers normally destabilize presynaptic nerve terminals during neuromuscular synaptogenesis. And, this negative and dynamic destabilizing factor(s) requires active nerve and, ultimately, muscle activity. Terminal Schwann cells play crucial roles to antagonize the destabilizing activity and thus to stabilize the NMJ. In the absence of Schwann cells, the destabilizing activity of such factors on presynaptic terminals is unopposed, which leads to synapse loss at the NMJ. In this scenario, blocking either nerve or muscle activity (as in *CRD-Nrg1*^*–/–*^*Snap25 ^–^*^/–^, *CRD-Nrg1*^*–/–*^*Chat^−^*^/–^, *CRD-Nrg1*^*–/–*^*Chrna1*^−/−^, *CRD-Nrg1*^*–/–*^*Cacnb1*^*–/–*^, or *CRD-Nrg1*^*–/–*^*Ryr1*^*–/–*^mice), would lead to a blockade of the muscle-derived destabilizing factor(s), which would allow Schwann cell deficient NMJs to form. Identification of the muscle-derived, muscle activity dependent factor(s) that regulate NMJ formation will provide important new insights into the mechanisms underlying neuromuscular synapse formation, maintenance and elimination.

A third, non-mutually exclusive possibility from the second possibility described above, is that Schwann cells may regulate synaptic activity itself. In this instance, the absence of Schwann cells would be predicted to lead to dysregulated synaptic activity. We have previously shown that spontaneous neuromuscular synaptic activity is markedly increased in *Cacnb1*^−/−^and *Ryr1*^−/−^mice, compared with control, likely due to precocious maturation of the NMJs in *Cacnb1*^−/−^and *Ryr1*^−/−^mice [68]. Remarkably, spontaneous synaptic activity is significantly further increased in Schwann cell deficient NMJs (*i*.*e*., in *CRD-Nrg1*^*–/–*^*Cacnb1*^*–/–*^and *CRD-Nrg1*^*–/–*^*Ryr1*^*–/–*^mice), compared with NMJs in *Cacnb1*^−/−^or *Ryr1*^−/−^mice, respectively. These further increases in spontaneous synaptic activity are likely due to the lack of Schwann cells at the NMJ in *CRD-Nrg1*^*–/–*^*Cacnb1*^*–/–*^and *CRD-Nrg1*^*–/–*^*Ryr1*^*–/–*^mice, suggesting that Schwann cells may regulate spontaneous synaptic activity during NMJ synaptogenesis. We do not know how evoked synaptic transmission is affected at the NMJs in *CRD-Nrg1*^*–/–*^*Cacnb1*^*–/–*^and *CRD-Nrg1*^*–/–*^*Ryr1*^*–/–*^mice since we were unable to obtain recordings of evoked end-plate potentials from Schwann cell deficient mutant mice due to technical difficulties. During our electrophysiological recordings, we have made numerous attempts to apply suction electrodes to the phrenic nerves in order to deliver electrical stimulation to the nerves in *CRD-Nrg1*^*–/–*^*Cacnb1*^*–/–*^or *CRD-Nrg1*^*–/–*^*Ryr1*^*–/–*^mice. However, we were unable to visualize live, unstained phrenic nerves under the microscope in double mutant mice, due to the fact that the phrenic nerves appeared transparent in the absence of Schwann cells. Nevertheless, the marked increases in spontaneous synaptic activity in NMJs lacking Schwann cells (*i*.*e*., *CRD-Nrg1*^*–/–*^*Cacnb1*^*–/–*^versus *Cacnb1*^*–/–*^; and *CRD-Nrg1*^*–/–*^*Ryr1*^*–/–*^versus *Ryr1*^*–/–*^) support the possibility, namely that Schwann cells may protect developing neuromuscular synapses by negatively regulating synaptic activity. Thus, the regulation of synaptic activity by Schwann cells appears to be critical for NMJ formation during development. Consistent with this idea, a recent Schwann cell ablation study demonstrates that these cells continue to regulate synaptic activity postnatally (Barik et al., 2016). These studies of synapses lacking Schwann cells give added support to the studies of intact synapses that demonstrate that the activation of Schwann cells by neural activity regulates presynaptic as well as postsynaptic function [48]. Previous studies have shown that Schwann cells are required for the maintenance of presynaptic structure in developing NMJs [11, 14] and play important roles in synapse elimination [15, 87]. It is possible that Schwann cells may preserve the NMJs, at least in part, by regulating the levels of synaptic activity (and therefore regulate muscle activity).

The results of the current study show that the level of muscle activity is a key regulator of neuromuscular synapse formation–NMJs can be established even in the absence of Schwann cells if muscle activity mediated by DHPR/Ryr1 is blocked. Our data are consistent with *in vitro* studies by O’Brien et al., [88, 89], which show that excessive activity, either by the topical application of ACh and high [Ca^2+^] to immature neuromuscular synapses, or by continuous stimulation of the nerve or the muscle, leads to muscle denervation. Importantly, our findings demonstrate that a blockade of muscle activity, instead of neuronal activity, is the key to preserving the developing neuromuscular synapses. We show that neuromuscular synapses are established in the absence of Schwann cells when muscle activity is eliminated. These findings further suggest that skeletal muscle activity might destabilize developing presynaptic nerves and that Schwann cells play crucial roles in counteracting such a destabilizing activity to preserve neuromuscular synapses during development.

## Materials & methods

### Ethics statement

All experimental protocols followed National Institutes of Health Guidelines and were approved by the University of Texas Southwestern Institutional Animal Care and Use Committee. The APN approval number is 2015–101081.

### Mice

Single mutant mice used in this study were previously generated; these mutant mice include *CRD-Nrg1* (Nrg1^tm1Lwr^, MGI:1928831) [38], *Snap25* (Snap25^tm1Mcw^, MGI: 2180178) [50], *Chat* (Chat^tm1Fhg^, MGI:2450310) [60], *Chrna1* (Chrna1^tm1Klee^, MGI:4462390) [64], *Cacnb1* (Cacnb1^tm1Rgg^, MGI:2181804) [67, 68], and *Ryr1* (Ryr1^tm1Alle^, MGI:4887253) [90–92]. We generated double mutant mice by crossing heterozygote mice to generate compound heterozygote mice, and then bred compound heterozygote mice together to generate homozygous mutant mice. As with single mutants, all homozygous double mutant mice died perinatally, due to NMJ defects. Thus, analyses were performed on mouse embryos between E14.5-E18.5, staged by timed-mating. Embryonic day 0.5 (E0.5) marked the day when a vaginal plug was detected. Mouse embryos were collected by cesarean section from anesthetized pregnant mice. Table 1 summarizes the numbers of mouse embryos analyzed in this study.

**Table 1:** A summary of the numbers (N) of mice analyzed in this study.

Genotype	N
*CRD-Nrg1*^*+/±*^*Chat*^*+/±*^	13
*CRD-Nrg1*^*–/–*^*Chat*^*+/±*^	13
*CRD-Nrg1*^*+/±*^*Chat*^*–/–*^	10
*CRD-Nrg1*^*–/–*^*Chat*^*–/–*^	10
*CRD-Nrg1*^*+/±*^*Snap25*^*+/±*^	3
*CRD-Nrg1*^*–/–*^*Snap25*^*+/±*^	3
*CRD-Nrg1*^*+/±*^*Snap25*^*–/–*^	3
*CRD-Nrg1*^*–/–*^*Snap25*^*–/–*^	3
*CRD-Nrg1*^*+/±*^*Chrna1*^*+/±*^	3
*CRD-Nrg1*^*–/–*^*Chrna1*^*+/±*^	3
*CRD-Nrg1*^*+/±*^*Chrna1*^*–/–*^	3
*CRD-Nrg1*^*–/–*^*Chrna1*^*–/–*^	3
*CRD-Nrg1*^*+/±*^*Cacnb1*^*+/±*^	10
*CRD-Nrg1*^*–/–*^*Cacnb1*^*+/±*^	9
*CRD-Nrg1*^*+/±*^*Cacna1*^*–/–*^	9
*CRD-Nrg1*^*–/–*^*Cacnb1*^*–/–*^	10
*CRD-Nrg1*^*–/–*^*Cacnb1*^*–/–*^*HSA-Cacnb1*	3
*CRD-Nrg1*^*+/±*^*Ryr1*^*+/±*^	7
*CRD-Nrg1*^*–/–*^*Ryr1*^*+/±*^	6
*CRD-Nrg1*^*+/±*^*Ryr1*^*–/–*^	6
*CRD-Nrg1*^*–/–*^*Ryr1*^*–/–*^	7

### Light microscopy

#### Morphological analyses of the NMJs

Analyses were performed using procedures described previously [93, 94]. Briefly, skeletal muscles were fixed in 2% paraformaldehyde, rinsed in sodium phosphate buffer saline (PBS) for 90 minutes and incubated in 100 mM glycine in PBS for 30 minutes. Muscle samples were then incubated in a blocking buffer containing 500 mM NaCl, 0.01 M phosphate buffer, 1% BSA and 0.01% thimerosal for 45 minutes. AChRs were detected using either Texas red-, or Alexa Fluor 647- conjugated α-bungarotoxin (α-bgt) (2 nM, Molecular Probes). For double-labeling experiments, we used Texas Red-conjugated α-bungarotoxin (α-bgt) (2 nM, Molecular Probes) for AChRs and the following primary antibodies for pre-synaptic nerve/nerve terminals: anti-NF150 (neurofilament protein (1:1000, Chemicon, Temecula, CA), anti-synaptotagmin 2 (Syt2) (1:1000) [95], anti-syntaxin 1 (1:1000, generous gifts from Dr. Thomas Südhof, Stanford University School of Medicine, Palo Alto, CA), followed by fluorescein(FITC)-conjugated goat anti-rabbit IgG secondary antibodies (code# 111-095-144, Jackson ImmunoResearch). For triple-labeling experiments, we used rabbit anti-S100β (1:500, Dako, Carpinteria, CA) to label Schwann cells and mouse anti-neurofilament H non-phosphorylated (1:500, SMI-32, abcam, Cambridge, MA) to label nerves, and Alexa Fluor 647-conjugated α-bungarotoxin (α-bgt) (2 nM, Molecular Probes) to label AChRs, followed by fluorescein(FITC)-conjugated goat anti-rabbit IgG (Code# 111-095-144, Jackson ImmunoResearch) and Texas red-conjugated goat anti-mouse IgG (Code# 115-075-146, Jackson ImmunoResearch). After secondary antibody incubation, muscle samples were washed extensively and mounted in anti-fade medium (Fluoro-Gel with Tris Buffer, diluted 1:1 with 50% glycerol, Electron Microscopy Sciences, Hatfield, PA). Images were acquired using a Zeiss LSM 510 confocal microscope.

#### Immunostaining of motor neuron

Cryostat sections (transverse section, 14 μm) were collected from cervical spinal segments of mouse embryos (E18.5). The sections were incubated with anti-choline transporter (CHT) antibodies [62] (polyclonal, 1:1000, generous gifts from Dr. Randy Blakely, Vanderbilt University, Nashville, Tennessee), followed by fluorescein (FITC)-conjugated goat anti-rabbit IgG secondary antibodies (#111-095-144, Jackson ImmunoResearch). Images were acquired using a Zeiss LSM 880 confocal microscope.

### Electrophysiology

E18.5 diaphragm muscles from mutant mice and their littermate controls were used for intracellular recording as previously described [94, 96]. Diaphragm muscles with phrenic nerves attached were acutely isolated in oxygenated (95% O_2_, 5% CO_2_) Ringer’s solution (136.8 mM NaCl, 5 mM KCl, 12 mM NaHCO_3_, 1 mM NaH_2_PO_4_, 1 mM MgCl_2_, 2 mM CaCl_2_, and 11 mM d-glucose, pH 7.3) [97]. End-plate regions were identified under a water-immersion objective (Olympus BX51 WI) and impaled with glass micropipettes filled with 2 M potassium citrate and 10 mM potassium chloride (resistance 20–40 MΩ). Miniature end-plate potentials (mEPPs) were acquired using an intracellular amplifier (AxoClamp-2B) and digitized with a Digidata 1332 (Molecular Devices, Sunnyvale, CA, USA). Data were analyzed with Mini Analysis Program (Synaptosoft, Inc., Decatur, GA).

### Electron microscopy

Diaphragm muscles with phrenic nerves attached were dissected in Ringer’s solution from mouse embryos at E18.5. Samples were then fixed with 1% glutaraldehyde in 0.1 M phosphate buffer (pH 7.4) and kept in the same fixative overnight at 4°C. After a rinse in 0.1 M phosphate buffer, diaphragm muscles were trimmed into small pieces. Tissues were then post-fixed with 1% osmium tetroxide for 3 hr. on ice, dehydrated through a graded series of ethanol, infiltrated, and polymerized in Epon 812 (Polysciences, Warrington, PA, USA). Prior to embedding in Epon, the phrenic nerve trunks were cut half-way from the muscles, and the nerve trunks were embedded separately. These phrenic nerves were later cross sectioned for EM. Ultrathin sections (70 nm) were prepared and mounted on Formvar-coated grids, then stained with uranyl acetate and lead citrate. Electron micrographs were acquired using a Tecnai electron microscope (Netherlands) operated at 120 kV.

### Statistical analysis

End-plate sizes were measured using ImageJ (NIH), with the measurements made blind with respect to the genotypes (at least 3 mice were analyzed in each group). Raw data were pooled and calculated as the mean ± standard error of the mean (SEM). Sigma Plot (version 11.0) was used to analyze statistics. Statistical differences among multiple groups were determined using one-way analysis of variance (ANOVA), followed by a Tukey post hoc test for pairwise multiple comparison between groups.

## Supporting information

S1 Fig Rescue of diaphragm muscle denervation in *CRD-Nrg1*^*–/–*^*Chat*^*−/−*^mice (E16.5). Embryonic diaphragm muscles (E16.5) from the control (*CRD-Nrg1*^*+/+*^*Chat* ^*+/–*^) (A), *CRD-Nrg1*^*–/–*^*Chat* ^*+/–*^(B), *CRD-Nrg1*^*+/–*^*Chat*^*−/−*^(C) and *CRD-Nrg1*^*–/–*^*Chat*^*−/−*^(D) mice were immuno-stained by a mixture of antibodies (anti-NF150 and anti-synaptotagmin 2) to reveal innervation pattern (green). Low power images show the innervation pattern of the entire diaphragm muscles. The phrenic nerves innervate the diaphragm muscles bilaterally. The phrenic nerves are absent in *CRD-Nrg1*^*–/–*^*Chat* ^*+/–*^muscle (B); this lack of innervation is rescued in *CRD-Nrg1*^*–/–*^*Chat*^*−/−*^muscle (D). Note that the nerves are highly defasciculated in *CRD-Nrg1*^*–/–*^*Chat*^*−/−*^muscle. Scale bar: A-D: 500 μm.
(TIF)

S2 Fig Motor neuron staining and motor axon counts in *CRD-Nrg1*^*–/–*^*Chat*^*−/−*^mice (E18.5). **A:** Cross sections of cervical spinal cords were immune-stained by anti-CHT antibodies, which label motor neurons (arrow) in the ventral horn of the spinal cord (upper panels show low-power images of entire spinal cords, and lower panels show high-power views of ventral horn). **B**: Low-power EM images of the phrenic nerve trunk (cross section) showing individual axons within the nerve trunk. **C**: Quantification of motor axons. The average motor axon numbers per phrenic nerve are similar between control (248 ± 8, N = 3 mice) and *CRD-Nrg1*^*–/–*^*Chat*^*−/−*^(244 ± 11, N = 3 mice).
(TIF)

S3 Fig Amplitude distribution of mEPPs. The amplitudes were plotted against either event numbers (upper panels) or percentage of total events (lower panels). In both control and mutant mice, the amplitude distribution patterns exhibited a single peak, with a right-skewed tail. The mEPP frequencies were massively increased in mutants [*Cacnb1*^*–/–*^, 3 mice, 39 cells, 1919 events (**B**); *CRD-Nrg1*^*–/–*^*Cacnb1*^*–/–*^, 3 mice, 42 cells, 8621 events (**C**); *Ryr1*^*–/–*^, 4 mice, 36 cells, 1726 events (**D**) and *CRD-Nrg1*^*–/–*^*Ryr1*^*–/–*^ 3 mice, 41 cells, 4707 events (**E**)], compared with the control [8 mice, 92 cells, 329 events (**A**)].
(TIF)

## References

[1] Ko CP, Robitaille R. Perisynaptic Schwann Cells at the Neuromuscular Synapse: Adaptable, Multitasking Glial Cells. Cold Spring Harb Perspect Biol. 2015;7(10):a020503 10.1101/cshperspect.a020503 . 26430218 PMC4588062

[2] Li L, Xiong WC, Mei L. Neuromuscular Junction Formation, Aging, and Disorders. Annu Rev Physiol. 2018;80:159–88. Epub 2017/12/02. 10.1146/annurev-physiol-022516-034255 . 29195055

[3] Tintignac LA, Brenner HR, Ruegg MA. Mechanisms Regulating Neuromuscular Junction Development and Function and Causes of Muscle Wasting. Physiol Rev. 2015;95(3):809–52. Epub 2015/06/26. 10.1152/physrev.00033.2014 . 26109340

[4] Shi L, Fu AK, Ip NY. Molecular mechanisms underlying maturation and maintenance of the vertebrate neuromuscular junction. Trends Neurosci. 2012;35(7):441–53. Epub 2012/05/29. 10.1016/j.tins.2012.04.005 . 22633140

[5] Auld DS, Colomar A, Belair EL, Castonguay A, Pinard A, Rousse I, et al Modulation of neurotransmission by reciprocal synapse-glial interactions at the neuromuscular junction. J Neurocytol. 2003;32(5–8):1003–15. 10.1023/B:NEUR.0000020638.31068.9f WOS:000220340400040. 15034282

[6] Sugiura Y, Lin W. Neuron-glia interactions: the roles of Schwann cells in neuromuscular synapse formation and function. Biosci Rep. 2011;31(5):295–302. 10.1042/BSR20100107 . 21517783 PMC4573580

[7] Darabid H, Perez-Gonzalez AP, Robitaille R. Neuromuscular synaptogenesis: coordinating partners with multiple functions. Nature Reviews Neuroscience. 2014;15(11):703–18. 10.1038/nrn3821 WOS:000344083000007. 25493308

[8] Wu H, Xiong WC, Mei L. To build a synapse: signaling pathways in neuromuscular junction assembly. Development. 2010;137(7):1017–33. 10.1242/dev.038711 . 20215342 PMC2835321

[9] Burden SJ, Huijbers MG, Remedio L. Fundamental Molecules and Mechanisms for Forming and Maintaining Neuromuscular Synapses. Int J Mol Sci. 2018;19(2). Epub 2018/02/09. 10.3390/ijms19020490 29415504 PMC5855712

[10] Rochon D, Rousse I, Robitaille R. Synapse-glia interactions at the mammalian neuromuscular junction. J Neurosci. 2001;21(11):3819–29. WOS:000168957600017. 11356870 10.1523/JNEUROSCI.21-11-03819.2001 PMC6762689

[11] Barik A, Li L, Sathyamurthy A, Xiong WC, Mei L. Schwann Cells in Neuromuscular Junction Formation and Maintenance. J Neurosci. 2016;36(38):9770–81. Epub 2016/09/23. 10.1523/JNEUROSCI.0174-16.2016 27656017 PMC5030347

[12] Love FM, Thompson WJ. Schwann cells proliferate at rat neuromuscular junctions during development and regeneration. J Neurosci. 1998;18(22):9376–85. 9801376 10.1523/JNEUROSCI.18-22-09376.1998 PMC6792891

[13] Trachtenberg JT, Thompson WJ. Nerve terminal withdrawal from rat neuromuscular junctions induced by neuregulin and Schwann cells. J Neurosci. 1997;17(16):6243–55. . 9236235 10.1523/JNEUROSCI.17-16-06243.1997 PMC6568340

[14] Reddy LV, Koirala S, Sugiura Y, Herrera AA, Ko CP. Glial cells maintain synaptic structure and function and promote development of the neuromuscular junction in vivo. Neuron. 2003;40(3):563–80. . 14642280 10.1016/s0896-6273(03)00682-2

[15] Lee YI, Li Y, Mikesh M, Smith I, Nave KA, Schwab MH, et al Neuregulin1 displayed on motor axons regulates terminal Schwann cell-mediated synapse elimination at developing neuromuscular junctions. Proc Natl Acad Sci U S A. 2016;113(4):E479–87. Epub 2016/01/13. 10.1073/pnas.1519156113 26755586 PMC4743767

[16] Son Y-J, Thompson WJ. Schwann cell processes guide regeneration of pheripheral axons. Neuron. 1995;14:125–32. 7826630 10.1016/0896-6273(95)90246-5

[17] Son Y-J, Thompson WJ. Nerve sprouting in muscle is induced and guided by processes extended by Schwann cells. Neuron. 1995;14:133–41. 7826631 10.1016/0896-6273(95)90247-3

[18] Son Y-J, Trachtenberg JT, Thompson WJ. Schwann cells induce and guide sprouting and reinnervation of neuromuscular junctions. TINS. 1996;19(7):280–4. 10.1016/S0166-2236(96)10032-1 8799973

[19] Meyer D, Yamaai T, Garratt A, Riethmacher-Sonnenberg E, Kane D, Theill LE, et al Isoform-specific expression and function of neuregulin. Development. 1997;124(18):3575–86. . 9342050 10.1242/dev.124.18.3575

[20] Talmage DA, Role LW. Multiple personalities of neuregulin gene family members. J Comp Neurol. 2004;472(2):134–9. Epub 2004/03/30. 10.1002/cne.20091 15048682 PMC2367215

[21] Birchmeier C, Nave KA. Neuregulin-1, a key axonal signal that drives Schwann cell growth and differentiation. Glia. 2008;56(14):1491–7. Epub 2008/09/23. 10.1002/glia.20753 . 18803318

[22] Mei L, Xiong WC. Neuregulin 1 in neural development, synaptic plasticity and schizophrenia. Nat Rev Neurosci. 2008;9(6):437–52. Epub 2008/05/15. 10.1038/nrn2392 18478032 PMC2682371

[23] Fischbach GD, Aratake H, Corfas G, Falls DL, Goodearl A, Rosen KM. Trophic interactions at developing synapses Molecular Neurobiology: Mechanisms Common to Brain, Skin and Immune System: Wiley-Liss, Inc.; 1994 p. 173–90. 7724645

[24] Falls DL, Harris DA, Johnson FA, Morgan MM, Corfas G, Fishbach GD. M_r_ 42,000 ARIA: a protein that may regulate the accumulation of acetylcholine receptors at developing chick neuromuscular junctions. Cold Spring Harb Symp Quant Biol. 1990;15:397–406. 10.1101/sqb.1990.055.01.040 2132829

[25] Falls DL, Rosen KM, Corfas G, Lane WS, Fischbach GD. ARIA, a protein that stimulates acetylcholine receptor synthesis, is a member of the neu ligand family. Cell. 1993;72(5):801–15. . 8453670 10.1016/0092-8674(93)90407-h

[26] Ting AK, Chen Y, Wen L, Yin DM, Shen C, Tao Y, et al Neuregulin 1 promotes excitatory synapse development and function in GABAergic interneurons. J Neurosci. 2011;31(1):15–25. Epub 2011/01/07. 10.1523/JNEUROSCI.2538-10.2011 21209185 PMC3078582

[27] Chen YJ, Zhang M, Yin DM, Wen L, Ting A, Wang P, et al ErbB4 in parvalbumin-positive interneurons is critical for neuregulin 1 regulation of long-term potentiation. Proc Natl Acad Sci U S A. 2010;107(50):21818–23. Epub 2010/11/26. 10.1073/pnas.1010669107 21106764 PMC3003111

[28] Kwon OB, Longart M, Vullhorst D, Hoffman DA, Buonanno A. Neuregulin-1 reverses long-term potentiation at CA1 hippocampal synapses. J Neurosci. 2005;25(41):9378–83. 10.1523/JNEUROSCI.2100-05.2005 . 16221846 PMC6725708

[29] Jiang L, Emmetsberger J, Talmage DA, Role LW. Type III neuregulin 1 is required for multiple forms of excitatory synaptic plasticity of mouse cortico-amygdala circuits. J Neurosci. 2013;33(23):9655–66. Epub 2013/06/07. 10.1523/JNEUROSCI.2888-12.2013 23739962 PMC3865493

[30] Zhong C, Akmentin W, Du C, Role LW, Talmage DA. Axonal Type III Nrg1 Controls Glutamate Synapse Formation and GluA2 Trafficking in Hippocampal-Accumbens Connections. eNeuro. 2017;4(1). Epub 2017/03/10. 10.1523/ENEURO.0232-16.2017 28275713 PMC5329619

[31] Zhong C, Du C, Hancock M, Mertz M, Talmage DA, Role LW. Presynaptic type III neuregulin 1 is required for sustained enhancement of hippocampal transmission by nicotine and for axonal targeting of alpha7 nicotinic acetylcholine receptors. J Neurosci. 2008;28(37):9111–6. 10.1523/JNEUROSCI.0381-08.2008 . 18784291 PMC2754770

[32] Falls DL. Neuregulins and the neuromuscular system: 10 years of answers and questions. J Neurocytol. 2003;32(5–8):619–47. Epub 2004/03/23. 10.1023/B:NEUR.0000020614.83883.be:NEUR.0000020614.83883.be. . 15034257

[33] Vullhorst D, Ahmad T, Karavanova I, Keating C, Buonanno A. Structural Similarities between Neuregulin 1–3 Isoforms Determine Their Subcellular Distribution and Signaling Mode in Central Neurons. J Neurosci. 2017;37(21):5232–49. Epub 2017/04/23. 10.1523/JNEUROSCI.2630-16.2017 28432142 PMC5456106

[34] Buonanno A, Fischbach GD. Neuregulin and ErbB receptor signaling pathways in the nervous system. Curr Opin Neurobiol. 2001;11(3):287–96. Epub 2001/06/12. . 11399426 10.1016/s0959-4388(00)00210-5

[35] Wang J, Song F, Loeb JA. Neuregulin1 fine-tunes pre-, post-, and perisynaptic neuromuscular junction development. Dev Dyn. 2017;246(5):368–80. Epub 2017/03/01. 10.1002/dvdy.24494 28245533 PMC5910656

[36] Morano M, Ronchi G, Nicolo V, Fornasari BE, Crosio A, Perroteau I, et al Modulation of the Neuregulin 1/ErbB system after skeletal muscle denervation and reinnervation. Sci Rep. 2018;8(1):5047 Epub 2018/03/24. 10.1038/s41598-018-23454-8 29568012 PMC5864756

[37] Woldeyesus MT, Britsch S, Riethmacher D, Xu L, Sonnenberg-Riethmacher E, Abou-Rebyeh F, et al Peripheral nervous system defects in erbB2 mutants following genetic rescue of heart development. Genes Dev. 1999;13(19):2538–48. . 10521398 10.1101/gad.13.19.2538 PMC317072

[38] Wolpowitz D, Mason TB, Dietrich P, Mendelsohn M, Talmage DA, Role LW. Cysteine-rich domain isoforms of the neuregulin-1 gene are required for maintenance of peripheral synapses. Neuron. 2000;25(1):79–91. . 10707974 10.1016/s0896-6273(00)80873-9

[39] Lin W, Sanchez HB, Deerinck T, Morris JK, Ellisman M, Lee KF. Aberrant development of motor axons and neuromuscular synapses in erbB2-deficient mice. Proc Natl Acad Sci U S A. 2000;97(3):1299–304. . 10655525 10.1073/pnas.97.3.1299 PMC15603

[40] Erickson SL, O'Shea KS, Ghaboosi N, Loverro L, Frantz G, B M., et al ErbB3 is required for normal cerebellar and cardiac development: a comparison with ErbB2 and heregulin-deficient mice. Development. 1997;124:4999–5011. 9362461 10.1242/dev.124.24.4999

[41] Morris JK, Lin W, Hauser C, Marchuk Y, Getman D, Lee KF. Rescue of the cardiac defect in ErbB2 mutant mice reveals essential roles of ErbB2 in peripheral nervous system development. Neuron. 1999;23(2):273–83. . 10399934 10.1016/s0896-6273(00)80779-5

[42] Riethmacher D, Sonnenberg-Riethmacher E, Brinkmann V, Yamaai T, Lewin GR, Birchmeier C. Severe neuropathies in mice with targeted mutations in the ErbB3 receptor. Nature. 1997;389(6652):725–30. 10.1038/39593 . 9338783

[43] Yang X, Arber S, William C, Li L, Tanabe Y, Jessell TM, et al Patterning of muscle acetylcholine receptor gene expression in the absence of motor innervation. Neuron. 2001;30(2):399–410. . 11395002 10.1016/s0896-6273(01)00287-2

[44] Escher P, Lacazette E, Courtet M, Blindenbacher A, Landmann L, Bezakova G, et al Synapses form in skeletal muscles lacking neuregulin receptors. Science. 2005;308(5730):1920–3. 10.1126/science.1108258 . 15976301

[45] Marder E, Prinz AA. Modeling stability in neuron and network function: the role of activity in homeostasis. Bioessays. 2002;24(12):1145–54. Epub 2002/11/26. 10.1002/bies.10185 . 12447979

[46] Turrigiano G. Too many cooks? Intrinsic and synaptic homeostatic mechanisms in cortical circuit refinement. Annu Rev Neurosci. 2011;34:89–103. Epub 2011/03/29. 10.1146/annurev-neuro-060909-153238 . 21438687

[47] Auld DS, Robitaille R. Glial cells and neurotransmission: An inclusive view of synaptic function. Neuron. 2003;40(2):389–400. 10.1016/S0896-6273(03)00607-X WOS:000185875800014. 14556716

[48] Robitaille R. Modulation of synaptic efficacy and synaptic depression by glial cells at the frog neuromuscular junction. Neuron. 1998;21(4):847–55. 10.1016/S0896-6273(00)80600-5 WOS:000076697300027. 9808470

[49] Darabid H, Arbour D, Robitaille R. Glial Cells Decipher Synaptic Competition at the Mammalian Neuromuscular Junction. J Neurosci. 2013;33(4):1297–313. 10.1523/JNEUROSCI.2935-12.2013 WOS:000313956900003. 23345206 PMC6618718

[50] Washbourne P, Thompson PM, Carta M, Costa ET, Mathews JR, Lopez-Bendito G, et al Genetic ablation of the t-SNARE SNAP-25 distinguishes mechanisms of neuroexocytosis. Nat Neurosci. 2002;5(1):19–26. 10.1038/nn783 . 11753414

[51] Jahn R, Lang T, Sudhof TC. Membrane fusion. Cell. 2003;112(4):519–33. . 12600315 10.1016/s0092-8674(03)00112-0

[52] Wickner W, Schekman R. Membrane fusion. Nat Struct Mol Biol. 2008;15(7):658–64. 18618939 10.1038/nsmb.1451 PMC2488960

[53] Jahn R, Scheller RH. SNAREs—engines for membrane fusion. Nat Rev Mol Cell Biol. 2006;7(9):631–43. Epub 2006/08/17. 10.1038/nrm2002 . 16912714

[54] Sudhof TC, Rothman JE. Membrane fusion: grappling with SNARE and SM proteins. Science. 2009;323(5913):474–7. 10.1126/science.1161748 19164740 PMC3736821

[55] Rizo J, Sudhof TC. The membrane fusion enigma: SNAREs, Sec1/Munc18 proteins, and their accomplices—guilty as charged? Annu Rev Cell Dev Biol. 2012;28:279–308. Epub 2012/10/13. 10.1146/annurev-cellbio-101011-155818 . 23057743

[56] Tsim KW, Choi RC, Siow NL, Cheng AW, Ling KK, Jiang JX, et al ATP induces post-synaptic gene expressions in vertebrate skeletal neuromuscular junctions. J Neurocytol. 2003;32(5–8):603–17. Epub 2004/03/23. 10.1023/B:NEUR.0000020613.25367.78 . 15034256

[57] Choi RC, Siow NL, Cheng AW, Ling KK, Tung EK, Simon J, et al ATP acts via P2Y1 receptors to stimulate acetylcholinesterase and acetylcholine receptor expression: transduction and transcription control. J Neurosci. 2003;23(11):4445–56. Epub 2003/06/14. . 12805285 10.1523/JNEUROSCI.23-11-04445.2003 PMC6740789

[58] O'Malley JP, Moore CT, Salpeter MM. Stabilization of acetylcholine receptors by exogenous ATP and its reversal by cAMP and calcium. J Cell Biol. 1997;138(1):159–65. Epub 1997/07/14. 9214389 10.1083/jcb.138.1.159 PMC2139944

[59] Burnstock G. Historical review: ATP as a neurotransmitter. Trends Pharmacol Sci. 2006;27(3):166–76. Epub 2006/02/21. 10.1016/j.tips.2006.01.005 . 16487603

[60] Brandon EP, Lin W, D'Amour KA, Pizzo DP, Dominguez B, Sugiura Y, et al Aberrant patterning of neuromuscular synapses in choline acetyltransferase-deficient mice. J Neurosci. 2003;23(2):539–49. . 12533614 10.1523/JNEUROSCI.23-02-00539.2003 PMC6741871

[61] Misgeld T, Burgess RW, Lewis RM, Cunningham JM, Lichtman JW, Sanes JR. Roles of neurotransmitter in synapse formation: development of neuromuscular junctions lacking choline acetyltransferase. Neuron. 2002;36(4):635–48. . 12441053 10.1016/s0896-6273(02)01020-6

[62] Ferguson SM, Savchenko V, Apparsundaram S, Zwick M, Wright J, Heilman CJ, et al Vesicular localization and activity-dependent trafficking of presynaptic choline transporters. J Neurosci. 2003;23(30):9697–709. Epub 2003/10/31. . 14585997 10.1523/JNEUROSCI.23-30-09697.2003 PMC6740902

[63] Bowman WC, Prior C, Marshall IG. Presynaptic receptors in the neuromuscular junction. Ann N Y Acad Sci. 1990;604:69–81. Epub 1990/01/01. . 1977361 10.1111/j.1749-6632.1990.tb31983.x

[64] An MC, Lin W, Yang J, Dominguez B, Padgett D, Sugiura Y, et al Acetylcholine negatively regulates development of the neuromuscular junction through distinct cellular mechanisms. Proc Natl Acad Sci U S A. 2010;107(23):10702–7. 10.1073/pnas.1004956107 20498043 PMC2890820

[65] Franzini-Armstrong C, Jorgensen AO. Structure and development of E-C coupling units in skeletal muscle. Annu Rev Physiol. 1994;56:509–34. 10.1146/annurev.ph.56.030194.002453 . 8010750

[66] Tsien RW, Tsien RY. Calcium channels, stores, and oscillations. Annu Rev Cell Biol. 1990;6:715–60. 10.1146/annurev.cb.06.110190.003435 . 2177344

[67] Gregg RG, Messing A, Strube C, Beurg M, Moss R, Behan M, et al Absence of the beta subunit (cchb1) of the skeletal muscle dihydropyridine receptor alters expression of the alpha 1 subunit and eliminates excitation-contraction coupling. Proc Natl Acad Sci U S A. 1996;93(24):13961–6. . 8943043 10.1073/pnas.93.24.13961 PMC19477

[68] Chen F, Liu Y, Sugiura Y, Allen PD, Gregg RG, Lin W. Neuromuscular synaptic patterning requires the function of skeletal muscle dihydropyridine receptors. Nat Neurosci. 2011;14(5):570–7. 10.1038/nn.2792 21441923 PMC3083454

[69] Endo M. Calcium release from the sarcoplasmic reticulum. Physiol Rev. 1977;57(1):71–108. Epub 1977/01/01. 10.1152/physrev.1977.57.1.71 . 13441

[70] Martonosi AN. Mechanisms of Ca2+ release from sarcoplasmic reticulum of skeletal muscle. Physiol Rev. 1984;64(4):1240–320. Epub 1984/10/01. 10.1152/physrev.1984.64.4.1240 . 6093162

[71] Fleischer S, Inui M. Biochemistry and biophysics of excitation-contraction coupling. Annu Rev Biophys Biophys Chem. 1989;18:333–64. Epub 1989/01/01. 10.1146/annurev.bb.18.060189.002001 . 2660829

[72] Rios E, Brum G. Involvement of dihydropyridine receptors in excitation-contraction coupling in skeletal muscle. Nature. 1987;325(6106):717–20. 10.1038/325717a0 . 2434854

[73] Coronado R, Morrissette J, Sukhareva M, Vaughan DM. Structure and function of ryanodine receptors. Am J Physiol. 1994;266(6 Pt 1):C1485–504. 10.1152/ajpcell.1994.266.6.C1485 . 8023884

[74] Takeshima H, Nishimura S, Matsumoto T, Ishida H, Kangawa K, Minamino N, et al Primary structure and expression from complementary DNA of skeletal muscle ryanodine receptor. Nature. 1989;339(6224):439–45. Epub 1989/06/08. 10.1038/339439a0 . 2725677

[75] Otsu K, Willard HF, Khanna VK, Zorzato F, Green NM, MacLennan DH. Molecular cloning of cDNA encoding the Ca2+ release channel (ryanodine receptor) of rabbit cardiac muscle sarcoplasmic reticulum. J Biol Chem. 1990;265(23):13472–83. Epub 1990/08/15. . 2380170

[76] Zorzato F, Fujii J, Otsu K, Phillips M, Green NM, Lai FA, et al Molecular cloning of cDNA encoding human and rabbit forms of the Ca2+ release channel (ryanodine receptor) of skeletal muscle sarcoplasmic reticulum. J Biol Chem. 1990;265(4):2244–56. Epub 1990/02/05. . 2298749

[77] Nakai J, Imagawa T, Hakamat Y, Shigekawa M, Takeshima H, Numa S. Primary structure and functional expression from cDNA of the cardiac ryanodine receptor/calcium release channel. FEBS Lett. 1990;271(1–2):169–77. Epub 1990/10/01. . 2226801 10.1016/0014-5793(90)80399-4

[78] Hakamata Y, Nakai J, Takeshima H, Imoto K. Primary structure and distribution of a novel ryanodine receptor/calcium release channel from rabbit brain. FEBS Lett. 1992;312(2–3):229–35. Epub 1992/11/09. . 1330694 10.1016/0014-5793(92)80941-9

[79] Furuichi T, Furutama D, Hakamata Y, Nakai J, Takeshima H, Mikoshiba K. Multiple types of ryanodine receptor/Ca2+ release channels are differentially expressed in rabbit brain. J Neurosci. 1994;14(8):4794–805. . 8046450 10.1523/JNEUROSCI.14-08-04794.1994 PMC6577160

[80] Giannini G, Clementi E, Ceci R, Marziali G, Sorrentino V. Expression of a ryanodine receptor-Ca2+ channel that is regulated by TGF-beta. Science. 1992;257(5066):91–4. . 1320290 10.1126/science.1320290

[81] Dennis MJ, Ziskind-Conhaim L, Harris AJ. Development of neuromuscular junctions in rat embryos. Dev Biol. 1981;81(2):266–79. . 7202841 10.1016/0012-1606(81)90290-6

[82] Bennett MR, Pettigrew AG. The formation of synapses in striated muscle during development. J Physiol. 1974;241(2):515–45. . 4443927 10.1113/jphysiol.1974.sp010670 PMC1331046

[83] Oppenheim RW, Prevette D, D'Costa A, Wang S, Houenou LJ, McIntosh JM. Reduction of neuromuscular activity is required for the rescue of motoneurons from naturally occurring cell death by nicotinic-blocking agents. J Neurosci. 2000;20(16):6117–24. . 10934261 10.1523/JNEUROSCI.20-16-06117.2000 PMC6772570

[84] Dahm LM, Landmesser LT. The regulation of intramuscular nerve branching during normal development and following activity blockade. Dev Biol. 1988;130(2):621–44. . 3058544 10.1016/0012-1606(88)90357-0

[85] Dahm LM, Landmesser LT. The regulation of synaptogenesis during normal development and following activity blockade. J Neurosci. 1991;11(1):238–55. . 1898747 10.1523/JNEUROSCI.11-01-00238.1991 PMC6575191

[86] Gould TW, Oppenheim RW. Motor neuron trophic factors: therapeutic use in ALS? Brain Res Rev. 2011;67(1–2):1–39. Epub 2010/10/26. 10.1016/j.brainresrev.2010.10.003 20971133 PMC3109102

[87] Smith IW, Mikesh M, Lee Y, Thompson WJ. Terminal Schwann cells participate in the competition underlying neuromuscular synapse elimination. J Neurosci. 2013;33(45):17724–36. Epub 2013/11/08. 10.1523/JNEUROSCI.3339-13.2013 24198364 PMC3818548

[88] O'Brien RA, Ostberg AJ, Vrbova G. The effect of acetylcholine on the function and structure of the developing mammalian neuromuscular junction. Neuroscience. 1980;5(7):1367–79. Epub 1980/01/01. . 7402476 10.1016/0306-4522(80)90209-2

[89] O'Brien RA, Ostberg AJ, Vrbova G. Protease inhibitors reduce the loss of nerve terminals induced by activity and calcium in developing rat soleus muscles in vitro. Neuroscience. 1984;12(2):637–46. Epub 1984/06/01. . 6379504 10.1016/0306-4522(84)90079-4

[90] Barone V, Bertocchini F, Bottinelli R, Protasi F, Allen PD, Franzini Armstrong C, et al Contractile impairment and structural alterations of skeletal muscles from knockout mice lacking type 1 and type 3 ryanodine receptors. FEBS Lett. 1998;422(2):160–4. . 9489997 10.1016/s0014-5793(98)00003-9

[91] Nakai J, Dirksen RT, Nguyen HT, Pessah IN, Beam KG, Allen PD. Enhanced dihydropyridine receptor channel activity in the presence of ryanodine receptor. Nature. 1996;380(6569):72–5. Epub 1996/03/07. 10.1038/380072a0 . 8598910

[92] Buck ED, Nguyen HT, Pessah IN, Allen PD. Dyspedic mouse skeletal muscle expresses major elements of the triadic junction but lacks detectable ryanodine receptor protein and function. J Biol Chem. 1997;272(11):7360–7. . 9054435 10.1074/jbc.272.11.7360

[93] Liu Y, Oppenheim RW, Sugiura Y, Lin W. Abnormal development of the neuromuscular junction in Nedd4-deficient mice. Dev Biol. 2009;330(1):153–66. 10.1016/j.ydbio.2009.03.023 . 19345204 PMC2810636

[94] Liu Y, Padgett D, Takahashi M, Li H, Sayeed A, Teichert RW, et al Essential roles of the acetylcholine receptor gamma-subunit in neuromuscular synaptic patterning. Development. 2008;135(11):1957–67. 10.1242/dev.018119 18434415 PMC2650015

[95] Pang ZP, Melicoff E, Padgett D, Liu Y, Teich AF, Dickey BF, et al Synaptotagmin-2 is essential for survival and contributes to Ca2+ triggering of neurotransmitter release in central and neuromuscular synapses. J Neurosci. 2006;26(52):13493–504. 10.1523/JNEUROSCI.3519-06.2006 . 17192432 PMC6674714

[96] Sugiura Y, Chen F, Liu Y, Lin W. Electrophysiological characterization of neuromuscular synaptic dysfunction in mice. Methods Mol Biol. 2011;793:391–400. 10.1007/978-1-61779-328-8_26 . 21913115 PMC4590777

[97] Liley AW. An investigation of spontaneous activity at the neuromuscular junction of the rat. J Physiol. 1956;132(3):650–66. 13332600 10.1113/jphysiol.1956.sp005555 PMC1363576

