# Supplementary figures and images for: Blocking skeletal muscle DHPRs/Ryr1 prevents neuromuscular synapse loss in mutant mice deficient in type III Neuregulin 1 (CRD-Nrg1)
Source: PLoS Genet. 2019 Mar 14;15(3):e1007857. doi: 10.1371/journal.pgen.1007857 (PMC6417856; doi:10.1371/journal.pgen.1007857)

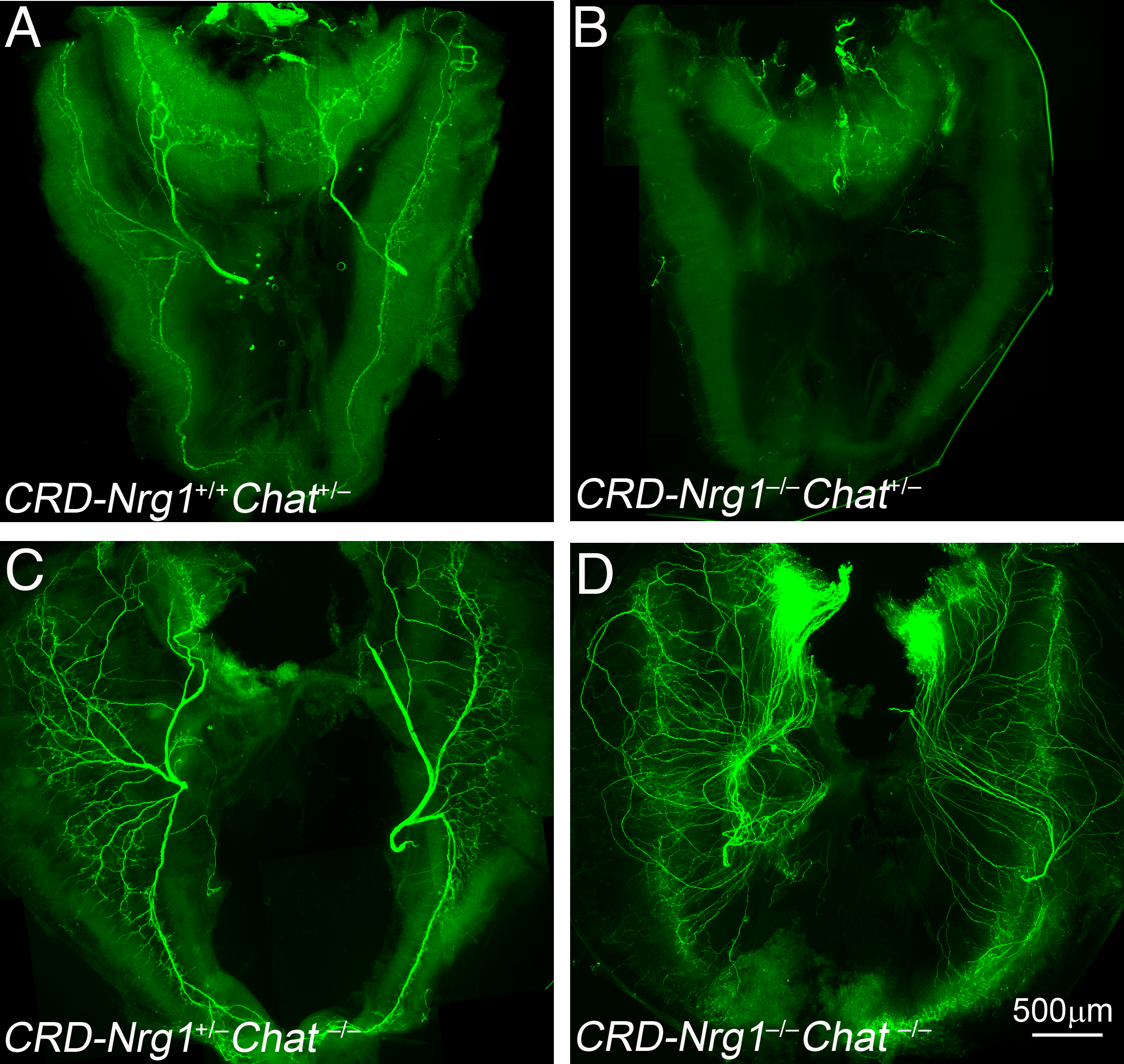

Supplement: S1 Fig — Embryonic diaphragm muscles (E16.5) from the control (CRD-Nrg1+/+Chat +/–) (A), CRD-Nrg1–/–Chat +/–(B), CRD-Nrg1+/–Chat−/−(C) and CRD-Nrg1–/–Chat−/−(D) mice were immuno-stained by a mixture of antibodies (anti-NF150 and anti-synaptotagmin 2) to reveal innervation pattern (green). Low power images show the innervation pattern of the entire diaphragm muscles. The phrenic nerves innervate the diaphragm muscles bilaterally. The phrenic nerves are absent in CRD-Nrg1–/–Chat +/–muscle (B); this lack of innervation is rescued in CRD-Nrg1–/–Chat−/−muscle (D). Note that the nerves are highly defasciculated in CRD-Nrg1–/–Chat−/−muscle. Scale bar: A-D: 500 μm. (TIF) [file pgen.1007857.s001.tif]

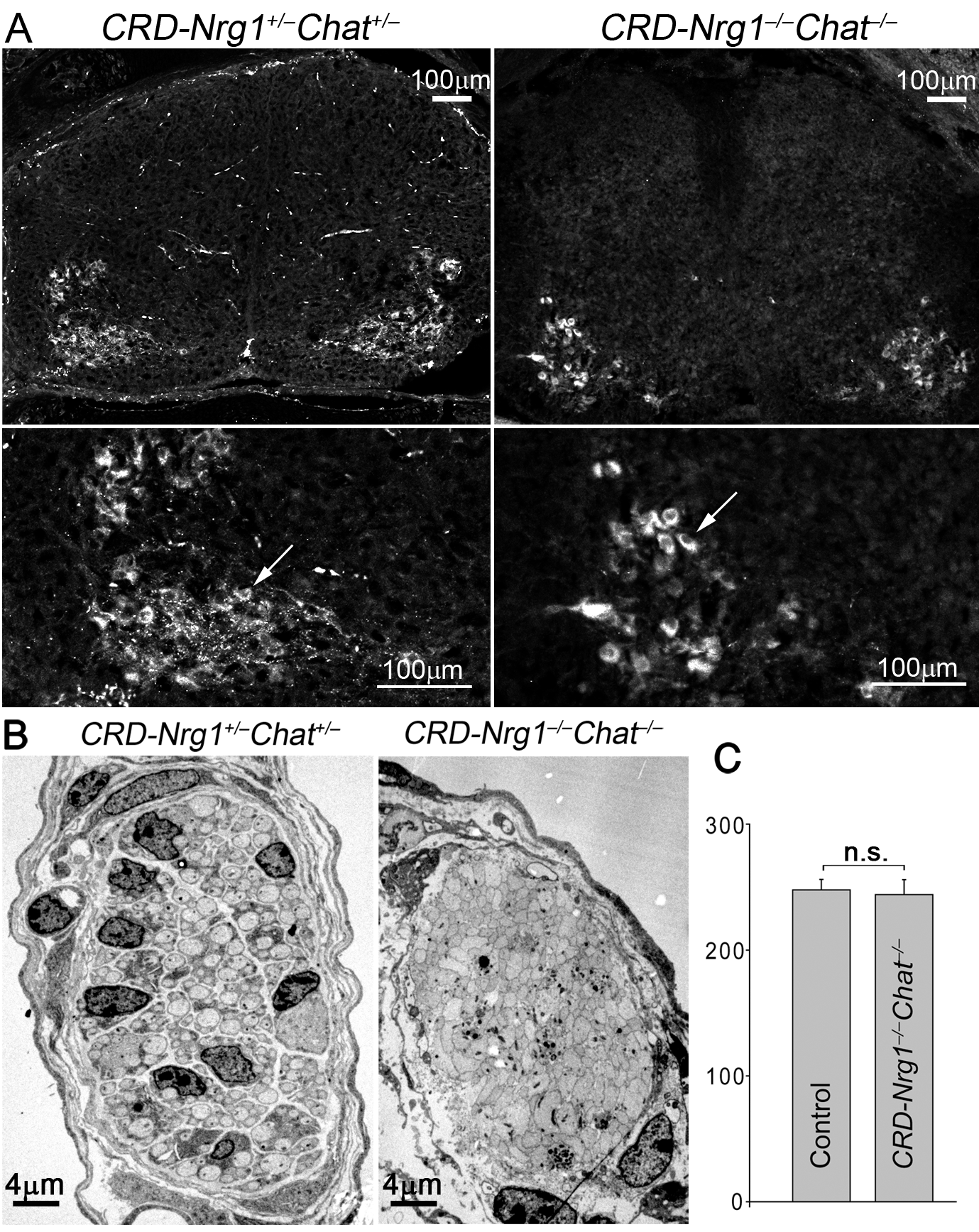

Supplement: S2 Fig — A: Cross sections of cervical spinal cords were immune-stained by anti-CHT antibodies, which label motor neurons (arrow) in the ventral horn of the spinal cord (upper panels show low-power images of entire spinal cords, and lower panels show high-power views of ventral horn). B: Low-power EM images of the phrenic nerve trunk (cross section) showing individual axons within the nerve trunk. C: Quantification of motor axons. The average motor axon numbers per phrenic nerve are similar between control (248 ± 8, N = 3 mice) and CRD-Nrg1–/–Chat−/−(244 ± 11, N = 3 mice). (TIF) [file pgen.1007857.s002.tif]

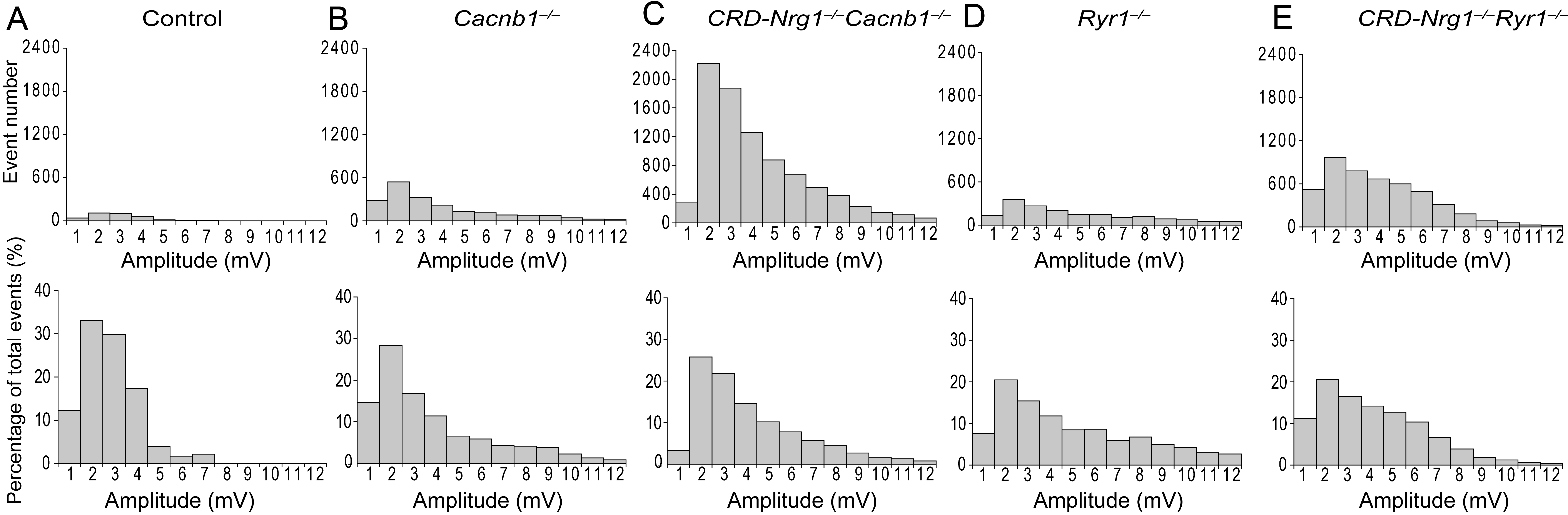

Supplement: S3 Fig — The amplitudes were plotted against either event numbers (upper panels) or percentage of total events (lower panels). In both control and mutant mice, the amplitude distribution patterns exhibited a single peak, with a right-skewed tail. The mEPP frequencies were massively increased in mutants [Cacnb1–/–, 3 mice, 39 cells, 1919 events (B); CRD-Nrg1–/–Cacnb1–/–, 3 mice, 42 cells, 8621 events (C); Ryr1–/–, 4 mice, 36 cells, 1726 events (D) and CRD-Nrg1–/–Ryr1–/– 3 mice, 41 cells, 4707 events (E)], compared with the control [8 mice, 92 cells, 329 events (A)]. (TIF) [file pgen.1007857.s003.tif]
